# Spatial-temporal analysis of natural hazards and disasters in the Greater Horn of Africa between 2010 and 2024 to inform disaster risk reduction, and surveillance and control strategies for climate and environmentally sensitive diseases

**DOI:** 10.1136/bmjopen-2025-104998

**Published:** 2025-11-04

**Authors:** Luke E Norris, Morgan Lemin, Louise A Kelly-Hope

**Affiliations:** 1 Institute of Infection, Veterinary and Ecological Sciences, University of Liverpool, Liverpool, UK

**Keywords:** Disease Outbreaks, Health informatics, Epidemics, Public health, Epidemiology

## Abstract

**Abstract:**

**Objective:**

To determine the spatial-temporal patterns of natural hazards and disasters in the Greater Horn of Africa, including climate and environmentally sensitive diseases, and compare the reporting consistencies across multiple open-access databases.

**Design:**

Cross-sectional retrospective secondary analysis of natural hazard and disaster data.

**Setting:**

Djibouti, Eritrea, Ethiopia, Kenya, Somalia, Sudan, South Sudan and Uganda.

**Data sources:**

Primary data from Emergency Events Database (EM-DAT), and comparative data from ReliefWeb, WHO Disease Outbreak News (WHO-DON), FloodList and Global Unique Disaster Identifier Number (GLIDE).

**Results:**

EM-DAT reported 228 natural hazards and disasters affecting 145.7 million people; highest numbers reported in Uganda (n=48), Kenya (n=46), Somalia (n=38) and Ethiopia (n=35); 175 geophysical, hydrological, meteorological and climatological hazards reported, including 118 floods, 26 droughts, 11 storms and 17 landslides; 46 epidemics reported, primarily bacterial (eg, cholera) or viral (eg, yellow fever, measles) diseases, with 20% preceded by a flood, drought or landslide within the previous 3 months. Reporting consistency and content varied considerably across the five databases.

**Conclusion:**

Natural hazards and disasters affect millions of people. There is an urgent need to improve database connectedness to facilitate better monitoring and mapping, which can inform disease forecasting and decision tools to develop preparedness and intervention strategies.

**STRENGTHS AND LIMITATIONS OF THIS STUDY**

- Comprehensive cross-sectional analysis of natural hazard events, disasters, infectious diseases and El Niño-Southern Oscillation relationships in Greater Horn of Africa using open-access data sources.
- Open-access natural hazard and disaster data sources provide a solid foundation for identifying frequency and magnitude of key events, patterns of high-risk places and times at a high level, and serve as a basis for more detailed investigations in endemic countries.
- Open-access natural hazards and disaster database sources vary in the type of information they collect and have several data gaps, including limited georeferenced information, which restricts comparability and opportunities for detailed mapping and analysis.

## Introduction

 The Greater Horn of Africa is a region particularly vulnerable to the impacts of climate change, an unrelenting major 21st century global health challenge, which has wide-ranging environmental consequences including warming temperatures, altered precipitation patterns and increased frequencies of extreme climate events.^1^,^3^ The Indian Ocean, adjacent to the region, is warming at the fastest rate among the global oceans and is considered to have increased the frequency and intensity of natural hazards, including cyclones, floods and droughts in the region.^4 5^ Such hazards can lead to disasters, for example, by increasing the risk of infectious disease outbreaks, food insecurity and widespread population displacement, which burden health systems and have negative economic and environmental consequences.^6^,^10^

Natural hazards may be further exacerbated by the El Niño-Southern Oscillation (ENSO), a major driver of large-scale climate variability across Africa, known to affect rainfall and temperature patterns. The ENSO fluctuates irregularly between El Niño (warm), La Niña (cold) and neutral phases and is shown to aggravate the impacts of droughts and floods across East Africa.^11^,^13^ There are many types of natural hazards that have been defined by the United Nations Office for Disaster Risk Reduction (UNDRR) with categories including biological, geological, hydro-meteorological and environmental.^10^

Understanding the spatial and temporal distributions of natural hazards and how they relate to disasters is important to inform disaster risk reduction, and disease surveillance and control strategies.^10^ The UNDRR aligns with and supports different global frameworks including the WHO Health Emergency and Disaster Risk Framework (WHO Health EDRM)^14^ and the Sendai Framework for Disaster Risk Reduction 2015–2023,^10^ which is connected with the Sustainable Development Goals (SDGs)^15^ and Paris Agreement.^16 17^

The UNDRR defines a disaster as “a serious disruption of the functioning of a community or a society at any scale due to hazardous events interacting with conditions of exposure, vulnerability and capacity, leading to one or more of the following: human, material, economic and environmental losses and impacts”.^10^ Standard definitions of natural hazards and disasters provide a constructive base that all organisations can use and adapt to suit their sectors, directives and policy objectives.

Globally, there are several databases that collate and store a range of data on natural hazards and related disasters, which can be used for programmatic and research purposes. The Emergency Events Database (EM-DAT) is widely cited in research and is a publicly available global disaster database, containing disaster events from 1900 to present.^18^ EM-DAT aligns with the UNDRR as it collects data on natural hazards and disasters with similar categories but has a different, yet complementary, focus. Additional open-source databases and resources include ReliefWeb,^19^ WHO Disease Outbreak News (WHO-DON)^20^ and FloodList,^21^ with the latter two comprising information primarily focused on disease outbreaks/epidemics and floods, respectively.

Analysing natural hazard and disaster databases is critical to understanding the trends and consequences of these events.^22 23^ Further, for disease control programmes, it is important to determine the relationship between different types of hazards and understand potential drivers of disease outbreaks and epidemics.^7 24^ However, making effective use of such databases is dependent on access to reliable and complete data across standardised reporting consistencies. This has been emphasised by the development of the Global Unique Disaster Identifier Number (GLIDE),^25^ which uses UNDRR terminology and provides a unique identifier to disasters to help link information from different sources. EM-DAT and ReliefWeb link with GLIDE, and a recent review between EM-DAT and an updated GLIDE version (GLIDEnumber V2.) has improved data cross-referencing; however, more efforts are still required to link all events.^26^

The overall aim of this study was to use open-access databases to analyse spatial-temporal distributions of natural hazards and disasters across eight countries of the Greater Horn of Africa between 2010 and 2024 to inform disaster risk reduction, and surveillance and control strategies for climate and environmentally sensitive diseases. Specifically, we (1) summarised the spatial-temporal distribution of natural hazards and disasters and number of people affected; (2) examined the temporal relationship between different hazard types with a focus on those preceding epidemics (biological hazards); (3) compared the content and reporting consistencies across multiple open-source databases and (4) summarised ENSO characteristics by country and in relation to the onset of different natural hazard types.

## Methods

### Study design

This retrospective secondary analysis of natural hazard and disaster data was conducted using a cross-sectional design and adhered to the Strengthening the Reporting of Observational Studies in Epidemiology (STROBE) guidelines (online supplemental file 1).^27^

### Study region

We focused our study on eight countries within the Greater Horn of Africa: Djibouti, Eritrea, Ethiopia, Kenya, Somalia, Sudan, South Sudan and Uganda. These countries are the focus of the Climate Sensitive Disease Forecasting Tool – CLIMSEDIS project^28^ and integrating spatial and temporal data on natural hazards and disasters will inform the tool’s development. Collectively, the countries are members of the Intergovernmental Authority on Development (IGAD),^29^ which is an entity across the region established to combat the impacts of famine and drought. The IGAD work in areas of agriculture and environment and feature a division for health and social development. All countries, except Eritrea, were also part of a WHO Health Emergency Appeal in 2024, highlighting the vulnerability to climate change in the region.^30^

### Natural hazards and disaster data sources

This study used five databases that record natural hazards and disasters. The open-access EM-DAT resource served as the ‘reference’ database for analysis across the study region.^18^ Briefly, EM-DAT compiles information that is broadly grouped as natural hazards and technological hazards. It is maintained by and distributed by the Centre for Research on the Epidemiology of Disasters (CRED). We used their natural hazard definitions and classifications, which are aligned with global frameworks, including the UNDRR,^10^ as shown in table 1.

**Table 1:** Summary of EM-DAT and UNDRR natural hazard classifications, definitions and examples

EM-DAT subgroup classification	EM-DAT hazard description	EM-DAT hazard type examples	UNDRR categories	UNDRR hazard description	UNDRR hazard examples
Biological	Caused by exposure to living organisms and/or their toxic substances or vector-borne diseases that they may carry	Epidemic Insect Infestation Animal accident	Biological	Of organic origin or conveyed by biological vectors	Epidemics Water-borne/ Vector-borne diseases Locust upsurge
Geophysical	Originating from solid earth	Earthquake Volcanic activity Mass movement (dry)	Geological	Originating from internal earth processes	Earthquake Volcanic eruptions Landslides
Hydrological	Caused by the occurrence, movement and distribution of surface and subsurface freshwater and saltwater	Flood Landslide Wave action	Hydro-meteorological	Related to water movement, distribution and water-induced events	Flood Flash floods Tsunamis
Meteorological	Caused by short-lived, micro-scale to meso-scale extreme weather and atmospheric conditions	Storms Tropical cyclones Extreme temperature	Hydro-meteorological	Caused by short-term atmospheric and weather phenomena	Cyclones Drought Extreme temperatures
Climatological*	Caused by long-lived, meso-scale to macro-scale atmospheric processes	Drought Wildfire	Environmental*	Arising from environmental degradation or ecological processes	Deforestation, Desertification, Land degradation

* Climatological classification is unique to EM-DAT, and Environmental category is unique to UNDRR.

EM-DAT, Emergency Events Database; UNDRR, United Nations Office for Disaster Risk Reduction.

EM-DAT records disasters that are classified by hazard types including biological, geophysical, hydrological, meteorological and climatological. A disaster is defined as “A situation or event which overwhelms local capacity, necessitating a request to the national or international level for external assistance; an unforeseen and often sudden event that causes great damage, destruction and human suffering”*,* which is more intrinsically related to emergency response thresholds than UNDRR. For a disaster to be listed in EM-DAT, it needs to meet at least one of the following criteria: (a) 10 or more people dead; (b) 100 or more people affected; (c) the declaration of a state of emergency and (d) a call for international assistance.

For selected analyses, biological hazards, including epidemics (subtypes including bacterial, parasitic and viral) and insect infestations were considered separately to the other natural hazard types including geophysical, hydrological, meteorological and climatological. EM-DAT’s data were compared with the following resources, each of which has a distinct focus and uses varying terminology.

- ReliefWeb: records information on natural hazards and disasters. Data listed on website.^19^
- WHO-DON: records primarily disease outbreak/epidemic information and associated natural hazards and disasters. Data listed on website.^20^
- FloodList: records flood events and summaries of news reports and articles. Data listed on website.^21^
- GLIDE: records natural hazards that are aligned with the UNDRR terminology and linked to a range of data sources. Data listed on website.^25^

### Data extraction and collation

First, data were downloaded from EM-DAT for the period of January 2010–September 2024, and compiled into a single datasheet, including information on the natural hazard classification and hazard type (table 1), start date, end date, country, location (Global Positioning System (GPS) and/or description), event name and the total number of people reported to be affected. Dataset used in this analysis is available through Dryad (dataset).^31^

Second**,** data for the same period from ReliefWeb,^19^ WHO-DON,^20^ FloodList^21^ and GLIDE^25^ were identified. Data from both ReliefWeb and WHO-DON were identified by searching the disaster entries for each country. Data compiled from ReliefWeb included links to the reports on natural hazards and/or disasters, many of which also reported a GLIDE unique identifier. Only the ReliefWeb reports in the ‘Disasters’ section of the website were used, as any disaster events mentioned within situation reports, appeals, or news and press releases were relatively difficult to search and collate relevant information. Data from WHO-DON included the links to the outbreak/epidemic information (biological hazard), which periodically described information on other natural hazard types (ie, geophysical, hydrological, meteorological and climatological). Data from FloodList included links to the flood events (hydrological hazard) that were found by screening the news reports for information in each country. Data from GLIDE included the GLIDE unique identifier. The ReliefWeb, WHO-DON and GLIDE datasets used in this analysis are available through Dryad (dataset).^31^

### EM-DAT spatial-temporal analysis

The spatial-temporal distribution of natural hazards and disasters and number of people affected across the eight countries were summarised. The χ² goodness-of-fit tests were performed using Microsoft Excel (Microsoft Corporation Version 16.89.1) to determine statistical differences (1) overall number of natural hazards and disasters by country; (2) overall number of natural hazards and disasters by year and month; (3) number of natural hazards (excluding biological) by type and (4) the number of epidemics (biological hazards) caused by a specific disease. In addition, the number of people affected was examined (1) by natural hazard (excluding biological) type and (2) by the specific disease. A p value <0.05 was considered statistically significant.

### Comparison of databases reporting and content

To cross-reference data entries from the different databases, first, the EM-DAT database was used as the ‘reference database’—ReliefWeb and GLIDE entries were compared across all natural hazards and disasters in EM-DAT; whereas, data within WHO-DON and FloodList were compared only to the outbreaks/epidemics and flood events in EM-DAT respectively as that was their primary focus.

Second, the ReliefWeb, WHO-DON and GLIDE databases were used as ‘reference databases’ separately to compare to the entries within the EM-DAT database. As above, ReliefWeb data were downloaded and compared with all disasters in EM-DAT, while the WHO-DON downloaded data were only compared with the epidemics in EM-DAT. FloodList was not used as a ‘reference database’ for comparison to EM-DAT due to the open format of the reports and significant effort required to format reports from this source into a data format. All GLIDE entries that were downloaded were compared with EM-DAT data. Datasets used in this analysis are available through Dryad (dataset).^31^ A schematic showing the approaches to database reporting comparisons is available in online supplemental file 2.

To compare reporting consistencies across the five databases, we identified data entries that were describing the same natural hazard or disaster, based on the type and location (district) of a given report, and if the starting date was no more than 3 months apart across both databases. This information was added to the compiled data. As a singular natural hazard or disaster report in one database could be considered the same as multiple data entries within another database, we calculated the proportion of hazards or disasters for each ‘reference database’ that could be considered the same event across all five database comparisons carried out.

To highlight the different content available in the databases, we presented five examples related to each of the biological, geophysical, hydrological, meteorological and climatological hazard categories.

### Temporal relationship between different hazard types

To explore the relationship between the EM-DAT different hazard types, with a focus on those preceding epidemics (biological hazards). First, we identified epidemics that were preceded by biological, geophysical, hydrological, meteorological and/or climatological hazards occurring in the same reported locations (district). Second, as a form of sensitivity analysis, we considered the transmission cycle durations of environmentally sensitive infectious diseases, which range from days to weeks depending on if they are vector-borne (eg, malaria, dengue) or environmentally mediated (eg, cholera).^6^,^9^ We examined the (1) type and number of hazards preceding epidemics and (2) number of people affected by epidemics, at three time periods based on transmission parameters, including (1) 1–3 months (optimal transmission period); 4–6 months (moderate/low transmission period) and 7–12 months (low/no transmission period). Assuming a non-normal distribution using a Shapiro-Wilk test, a two-tailed Mann-Whitney U test was performed using Microsoft Excel (Microsoft Corporation Version 16.89.1) to determine whether the distributions of the total number of people affected differed significantly between the 1–3-month time period and the combined transmission periods of 4–12 months. A p value<0.05 was considered statistically significant.

### ENSO characteristics in relation to natural hazards and disasters

Finally, to determine if the characteristics of the ENSO phases are linked to natural hazard or disaster onset month, we used the monthly Oceanic Nino Index (ONI) measure.^32^ The ONI is based on a 3-month running average of sea surface temperatures (SST) in the Niño 3.4 region (Pacific Ocean) developed by the National Oceanic and Atmospheric Administration (NOAA).^33^ Monthly ONI data were obtained from the Climate Protection Centre (CPC) provided by the NOAA.

ENSO phases included El Niño (warm), La Niña (cold) and neutral based on ONI measures (noted below), and we determined the ENSO phase, its duration (months) and the ONI range leading up to reported natural hazard or disasters.

1. El Niño phase: The Niño 3.4 region has an average SST at or above +0.5°C on the ONI.
2. La Niña phase: The Niño 3.4 region SST is at or below −0.5°C on the ONI.
3. Neutral phase: The Niño 3.4 region SST is between −0.5°C and +0.5°C on the ONI.

### Patient and public involvement

No patient and/or members of the public were included in this study.

## Results

### Summary of EM-DAT data

EM-DAT recorded information on 228 natural hazards or disasters across the eight countries with 175 (76.8%) reported as geophysical, hydrological, meteorological and climatological hazards, 46 (20.2%) as biological hazards including epidemics and 7 (3.1%) as insect infestations. The overall annual number of natural hazards or disasters ranged from 7 to 30 (median 14). Summaries are available in the online supplemental file 3.

Overall, there was a significant difference in the number of hazards by country when compared with a uniform distribution (χ²(7) = 74.3, p<0.001). The countries with the highest number of natural hazards or disasters overall were Uganda (n=48), Kenya (n=46), Somalia (n=38) and Ethiopia (n=35) (table 2).

**Table 2:** Summaries of EM-DAT total number of natural hazards of disasters for each country in (A) 3-year groupings and (B) quarterly groupings between January 2010 and September 2024

Total no. of natural hazards or disasters (3-year groupings)					
Country/year	2010–2012	2013–2015	2016–2018	2019–2021	2022–2024
Djibouti	1	0	1	3	1
Eritrea	0	0	0	1	0
Ethiopia	6	6	5	11	7
Kenya	11	9	10	9	7
Somalia	6	10	5	9	8
South Sudan	4	6	4	7	2
Sudan	6	5	7	10	3
Uganda	10	4	7	18	9
**Total**	**44**	**40**	**39**	**68**	**37**

Total no. of natural hazards or disasters per month (quarterly groupings)					
Country/month	Jan–Mar	Apr–Jun	July–Sep	Oct–Dec	Undefined start month*
Djibouti	1	3	0	2	0
Eritrea	1	0	0	0	0
Ethiopia	4	12	8	9	2
Kenya	16	14	2	13	1
Somalia	9	11	5	13	1
South Sudan	4	6	9	4	1
Sudan	1	8	17	0	5
Uganda	8	15	15	10	0
**Total**	**44**	**59**	**56**	**51**	**10**

Quarterly groupings=Q1 January–March, Q2 April–June, Q3 July–September, Q4 October–December. The annual and monthly data are available in online supplemental file 4.

* Natural hazards or disasters without a defined starting month are included in the ‘Undefined Start Month’ column.

EM-DAT, Emergency Events Database.

Overall, there was a significant difference in the number of hazards reported each year (when compared with a uniform distribution (χ²(14) = 32.3, p=0.004) but not monthly (χ²(11) = 18.9, p=0.063). The years with the highest number of natural hazards or disasters across the Horn of Africa countries were 2019 (n=30), 2020 (n=24), 2013 (n=19) and 2010 (n=18). The months with the highest number of natural hazards or disasters overall were April (n=29), August (n=26) and October (n=20). A total of 10 data entries did not have a defined start month within EM-DAT. Table 2 summarises multi-year and quarterly (January–March, April–June, July–September, October–December) trends. Annual and monthly data are available in the online supplemental file 4.

### Geophysical, hydrological, meteorological and climatological hazards

The countries with the highest number of EM-DAT geophysical, hydrological, meteorological and climatological hazards or disasters over the whole reporting period were Uganda (n=38), Kenya (n=37), Somalia (n=34) and Ethiopia (n=27).

**Figure 1:**
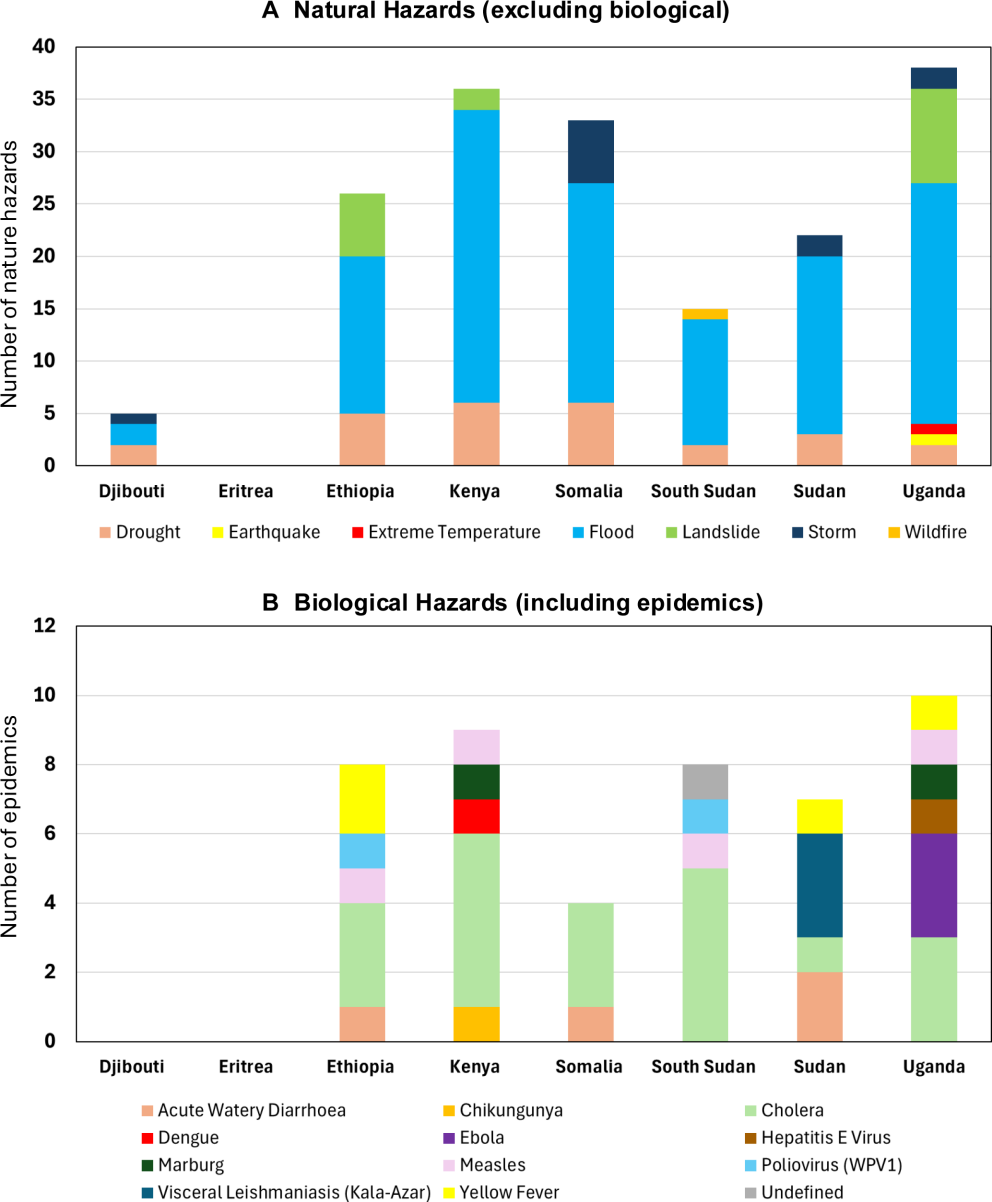
Summaries of EM-DAT data on the distribution of (A) natural hazards (excluding biological) and (B) biological hazard type (including epidemics) per country between January 2010 and September 2024. EM-DAT, Emergency Events Database.

Overall, there was a significant difference in the number of hazards by type of hazard when compared with a uniform distribution (χ²(6) = 425.5, p<0.001). The most common hazard types were floods (hydrological, n=118) and droughts (climatological, n=26) with fewer reported related to landslides (hydrological, n=17), storms (meteorological, n=11), earthquakes (geophysical, n=1), extreme temperatures (meteorological, n=1) and wildfires (climatological, n=1). The distribution of natural hazard type, excluding biological hazards, by each country is shown in figure 1A.

### Biological hazards

#### Epidemics

The countries with the highest number of EM-DAT epidemics (biological hazards) were Uganda (n=10), Kenya (n=9), Sudan (n=8), South Sudan (n=8) and Ethiopia (n=8). Overall, there was a significant difference in the number of epidemics caused by a specific disease when compared with a uniform distribution (χ²(10) = 71.6, p<0.001). The most common diseases were of cholera (n=20) followed by acute watery diarrhoea (n=4), measles (n=4) and yellow fever (n=4). Other epidemics included visceral leishmaniasis (n=3), Ebola (n=3), Marburg (n=2), poliovirus (WPV1) (n=2), chikungunya (n=1), dengue (n=1) and hepatitis E virus (n=1). Additionally, one epidemic listed for South Sudan did not provide a defined infectious disease. The distribution of epidemics by each country is shown in figure 1B.

#### Insect infestations

There were seven insect or locust infestations (biological hazard) reported in EM-DAT, which were reported across seven separate countries in 2019 and 2020, including Djibouti (n=1), Eritrea (n=1), Ethiopia (n=1), Kenya (n=1), Somalia (n=1), Sudan (n=1) and Uganda (n=1). South Sudan was the only country without a reported locust infestation in EM-DAT.

### Total number of people affected

Of the 228 natural hazards and disasters reported in EM-DAT, 203 also reported the number of people affected, with an overall total of 145 679 284. The country with the highest number of people affected by all natural hazards was Ethiopia (n=51 375 194), followed by Somalia (n=30 902 814), Kenya (n=25 045 090), Sudan (n=19 402 866), South Sudan (n=16 198 730), Uganda (n=2 152 422), Djibouti (n=602 168) and Eritrea (n=0).

**Figure 2:**
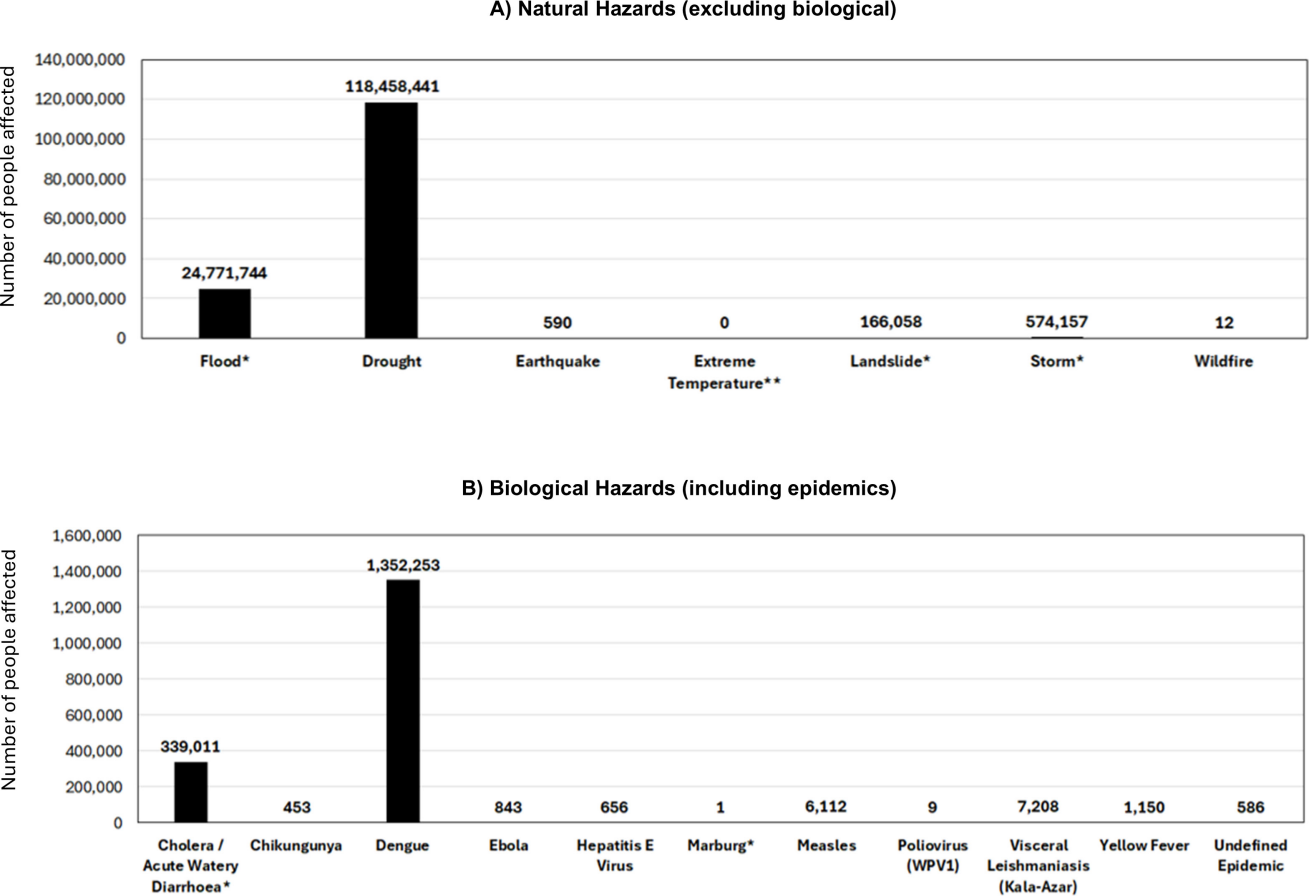
Summary of EM-DAT data showing (**A**) the total number of people affected by each natural hazard and (**B**) the total number of people affected by each epidemic between January 2010 and September 2024. *The number of people reported to be affected was not available for at least one natural hazard or disaster in this category. **The number of people affected was not available for any natural hazards or disaster in this category. EM-DAT, Emergency Events Database.

Overall, there was a significant difference in the number of people affected by natural hazard type when compared with a uniform distribution (χ²(6) = 568 861 564.9, p<0.001). The natural hazard types (excluding biological) that affected the most people were droughts (n=118 458 441) and floods (n=24 771 744) as shown in figure 2A. The most people affected by droughts were in Ethiopia (n=46 905 979), Somalia (n=22 835 264) and Kenya (n=20 150 000). The most people affected by floods were in Somalia (n=7 507 135), South Sudan (n=4 861 800) and Ethiopia (n=4 435 765).

Overall, there was a significant difference in the number of people affected by epidemics (biological hazard) caused by specific diseases when compared with a uniform distribution (χ²(10) = 10 549 354.61, p<0.001). The diseases that affected most people included dengue (n=1 352 253) and cholera (n=339 011) (figure 2B). The only country with people reported to be affected by dengue was Kenya (n=1 352 253) and the countries with the most people affected by cholera included Uganda (n=223 991), Somalia (n=35 675) and Sudan (n=31 904). There were no totals available for the number of people affected by any of the insect infestations reported in EM-DAT.

### Summary of ReliefWeb, WHO-DON and FloodList data

Data from ReliefWeb, WHO-DON and FloodList yielded a different set of natural hazards in comparison to EM-DAT. ReliefWeb contained fewer total entries (n=170) when compared with EM-DAT (n=228). Extractions from WHO-DON, which primarily focused on outbreaks/epidemics, yielded more entries (n=75) when compared with the epidemics listed in EM-DAT (n=46). FloodList reports comprised many more flood entries (n=224) than listed in EM-DAT (n=118). GLIDE reported more entries (n=250) across all countries except Eritrea than in EM-DAT (online supplemental file 3).

### Comparison of database reporting consistency

The first four database comparisons used EM-DAT as the reference database to compare to reports in ReliefWeb, WHO-DON, FloodList and GLIDE, separately (table 3). The comparisons revealed high reporting variation. Of the 228 total natural hazards or disasters reported in EM-DAT, 170 (74.6%) were considered the same disasters as reported by ReliefWeb, and 148 (65.4%) the same as reported in GLIDE. Of the 46 epidemics (biological hazards) listed in EM-DAT, only 12 (26.1%) could be linked to outbreak reports in WHO-DON. Of the 118 floods reported in EM-DAT, 51 (43.2%) could be linked to flood report articles in FloodList.

**Table 3:** Comparison of the five databases reporting on natural hazards and disasters

Comparison no.	Reference database	Database compared with	No. of natural hazards the same	% of natural hazards the same
1	EM-DAT	ReliefWeb	170	74.6%
2	EM-DAT	WHO-DON	12	26.1%
3	EM-DAT	FloodList	51	43.2%
4	EM-DAT	GLIDE	148	65.4%
5	ReliefWeb	EM-DAT	111	65.3%
6	WHO-DON	EM-DAT	29	36.7%
7	GLIDE	EM-DAT	130	52.0%

EM-DAT, Emergency Events Database; GLIDE, Global Unique Disaster Identifier Number; WHO-DON, WHO Disease Outbreak News.

Reversing the comparisons, similar levels of high variation were observed when comparing ReliefWeb, WHO-DON and GLIDE to EM-DAT. Of the 170 total hazards and disasters (natural and epidemics) reported in ReliefWeb, 111 (65.3%) were considered the same as those reported in EM-DAT. Of the 75 outbreaks reported in WHO-DON, only 29 (36.7%) could be considered the same epidemics as reported in EM-DAT. Of the 250 hazards and disasters reported in GLIDE, 130 (52.0%) were considered the same as those reported in EM-DAT.

### Comparison of database content of different hazard types

The five examples from the different hazards categories demonstrated the variability in reporting and access to additional related resources such as emergencies plans and links to other data sources.

1. Biological hazard example: EM-DAT: Kenya; Epidemic; Viral Disease; Dengue; Hazard number - 2021-0281-KEN; Location - Mombasa and Lamu Provinces; Start month/year - March 2021; Number of people affected - 1 352 253.
  - ReliefWeb: https://ReliefWeb.int/disaster/ep-2021-000047-ken
  - GLIDE: https://glidenumber.net/glide/public/search/details.jsp?glide=22169&record=2&last=105
2. Geophysical hazard example: EM-DAT Uganda; Earthquake; Hazard number - 2016-0329-UGA; Location - Minziro, Kanabulemu parish (Kakuuto district, Rakai province); Start month/year - September 2016; Number of people affected - 590. Note this is on the border with Tanzania and related links include both countries.
  - ReliefWeb: https://ReliefWeb.int/disaster/eq-2016-000098-tza
  - GLIDE: https://glidenumber.net/glide/public/search/details.jsp?glide=20744&record=1&last=1
3. Hydrological hazard example: EM-DAT: South Sudan; Flood; Hazard No. 2021-0481-SSD; Location - Mayendit County (Unity); Ayod, Fangak Counties (Jonglei), Northern Bahr el Ghazal, Upper Nile, Warrap, Western Equatoria states; Start month/year - May 2021; No. people affected - 835 000.
  - ReliefWeb: https://ReliefWeb.int/disaster/fl-2021-000108-ssd
  - FloodList: https://FloodList.com/africa/south-sudan-floods-august-2021
  - GLIDE: https://glidenumber.net/glide/public/search/details.jsp?glide=22231&record=1&last=46
4. Meteorological hazard example: EM-DAT: Somalia; Storm; Tropical Cyclone; Hazard number: 2018-0145-SOM; Location - Somaliland, Puntland; Start month/year - May 2018; No. people affected - 228 000.
  - ReliefWeb: https://ReliefWeb.int/disaster/tc-2018-000059-som
  - GLIDE: Number https://glidenumber.net/glide/public/search/details.jsp?glide=21068&record=1&last=1
5. Climatological hazard example: EM-DAT Ethiopia; Drought; Hazard number: 2015-9545-ETH; Location - Somali, Afar, Oromia, Amhara, Nations du Sud provinces; Start month/year - September 2015; No. people affected - 10 200 000.
  - ReliefWeb: https://reliefweb.int/disaster/dr-2015-000109-eth
  - GLIDE: https://glidenumber.net/glide/public/search/details.jsp?glide=20511&record=1&last=1

### Temporal relationships between different hazard types

Of the 46 epidemics (biological hazard) from the EM-DAT database, nine (19.6%) were identified as following a landslide (geophysical hazard), flood (hydrological hazard) or drought (climatological hazard) within the 3-month or optimal transmission period (table 4). These epidemics occurred across four countries, Ethiopia (n=3), Sudan (n=2), South Sudan (n=2) and Kenya (n=2). Five out of these nine epidemics were episodes of cholera, and six out of these nine epidemics followed a flood event. Overall, a total of 1 369 462 people were reported to be affected from the epidemics of cholera or acute watery diarrhoea (bacterial disease), yellow fever (arboviral disease) and viral poliovirus (viral disease) across the nine countries. The dengue outbreak in Kenya in 2020 following a drought reported the highest number of people affected (n=1 352 253).

**Table 4:** Summary of the EM-DAT natural hazard types (excluding biological) preceding epidemics (biological hazard) at three time periods between 2010 and 2024

Country	Proceeding hazard type* EM-DAT	Hazard type start date GLIDE reference†	Epidemic (biological hazard) EM-DAT	Epidemic start date GLIDE reference†	Common locations affected‡
3-month period					
Ethiopia	Flood	April 2013 FL-2013-000050-ETH	Yellow fever	July 2013 EP-2013-000067-ETH	SNNPR
Ethiopia	Landslide	October 2019 FL-2019-000116-ETH	Cholera	December 2019 GLIDE not available	SNNPR
Ethiopia	Flood	August 2022 FL-2022-000356-ETH	Cholera	September 2022 EP-2022-000323-ETH	Oromia
Kenya	Drought	January 2019 DR-2019-000024-KEN	Cholera	January 2019 EP-2019-000114-KEN	Garissa
Kenya	Drought	December 2020 DR-2014-000131-KEN	Dengue	March 2021 EP-2021-000047-KEN	Lamu
South Sudan	Flood	August 2013 FL-2013-000108-SSD	Poliovirus (WPV1)	October 2013 EP-2013-000137-SSD	Northern Bahr El Ghazal
South Sudan	Flood	July 2016 No GLIDE reference	Cholera	August 2016 EP-2016-000074-SSD	Al Wahdah State
Sudan	Flood	August 2016 FL-2016-000072-SDN	Acute Watery Diarrhoea	September 2016 EP-2017-000124-SDN	Blue Nile, Kassala
Sudan	Flood	July 2019 FL-2019-000064-SDN	Cholera	August 2019 EP-2019-000113-SDN	White Nile, Kassala, Khartoum
4–6-month period					
Kenya	Flood	April 2021 FL-2021-000038-KEN	Cholera	October 2022 EP-2022-000367-KEN	Tana River, Garissa, Marsabit, Homa Bay, Nairobi
Uganda	Flood	August 2011 FL-2011-000132-UGA	Cholera	January 2012 EP-2012-000031-UGA	Mbale, Sironko, Kasese
7–12-month period					
Kenya	Flood	May 2010 GLIDE not available	Measles	December 2010 GLIDE not available	Rift Valley
Kenya	Drought	January 2014 GLIDE not available	Cholera	December 2014 GLIDE not available	Mandera
Kenya	Flood	May 2017 GLIDE not available	Chikungunya	December 2017 GLIDE not available	Mombasa
South Sudan	Flood	August 2022 GLIDE not available	Cholera	February 2023 EP-2023-000039-SSD	Upper Nile
Sudan	Flood	August 2018 FL-2018-000128-SDN	Cholera	August 2019 EP-2019-000113-SDN	Kassala, Khartoum, Kordofan

* Preceding natural hazards and disaster start date listed as no more than 3 months prior to epidemic or in the same month if no start day is listed.

† GLIDE number included for cross-referencing.

‡ At least one common location (district) affected by both the epidemic and preceding natural hazard.

EM-DAT, Emergency Events Database; GLIDE, Global Unique Disaster Identifier Number; SNNPR, Southern Nations, Nationalities and Peoples' Region.

**Figure 3:**
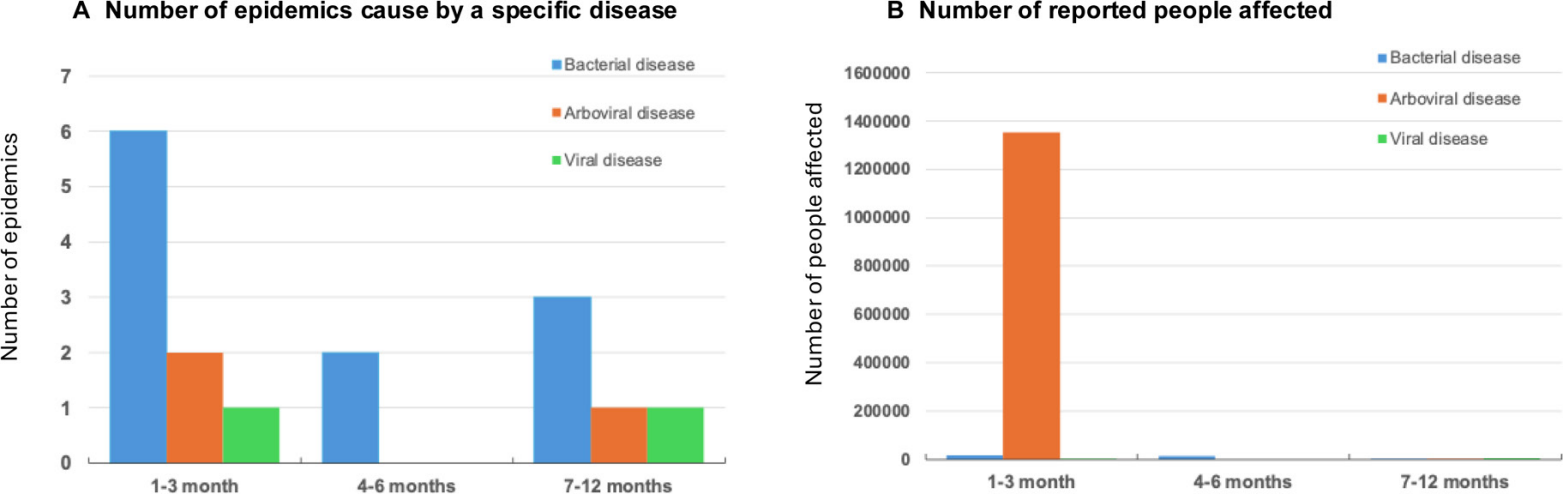
Summary of the EM-DAT reported (A) number and type of epidemics and (B) number of people affected from epidemics following a natural hazard at three time periods. EM-DAT, Emergency Events Database.

For the sensitivity analysis, when the 4–6-month and 7–12-month periods were compared, there were fewer reports of epidemics including cholera or acute watery diarrhoea (bacterial disease), chikungunya (arboviral disease) and measles (viral disease), and number of people affected including 12 977 and 5646 respectively. The results of the Mann-Whitney U test indicated that there was no significant difference in the distributions of the total number of people affected by epidemics between the 1–3-month transmission time period (median=781) and the 4–12-month transmission time period (median=1046), (U=27.5, p=1.00). Figure 3 summarises the epidemic (biological hazard) and the number of people reported to be affected across the time periods.

### ENSO phases link with disasters

The ENSO phases for each country were calculated for 218 of the 228 natural hazards with a defined start month. Overall, the ENSO neutral phase was the most common for natural hazard and disaster (excluding biological hazards) onset month (n=73), followed by the El Niño phase (n=57) and the La Niña phase (n=45). The ENSO neutral phase was also the most common for epidemic (biological hazard) onset month (n=17), followed by the La Niña phase (n=13) and the El Niño phase (n=12). A summary of ENSO data by country is available in online supplemental file 5.

Overall, there was no difference in the average length of the different ENSO phases preceding natural hazards and disasters (ie, El Niño phase 8 months; La Niña phase 8 months; and neutral phase 8 months). Table 5 summarises the average length of each ENSO phase (in months) preceding a natural hazard or disaster and the ONI range (minimum and maximum of the phase). The longest phases occurred for drought (neutral phase—average 17 months; ONI range −0.5 to −1.1), landslides (La Niña phase—average 15 months, ONI range −0.5 to −1.1), extreme temperature (El Niño phase—average 11 months, ONI range 0.5 to 1.9) and epidemics (neutral phase—average 11 months, ONI range 0.1 to 0.4).

**Table 5:** Summary of ENSO phase average length in months preceding natural hazards and disasters, and the ONI range for the El Niño, La Niña and neutral phases by natural hazard category and type

Hazard category	Hazard type	El Niño		La Niña		Neutral	
		No.	Average phase length (ONI range)	No.	Average phase length (ONI range)	No.	Average phase length (ONI range)
Biological	Epidemic	12	8 (0.5 to 2.6)	13	8 (−0.5 to −0.5)	18	11 (0.1 to 0.4)
	Insect infestation	7	4 (0.5 to 0.5)	–	–	–	–
Geophysical	Earthquake	–	–	1	2 (−0.5 to −0.6)	–	–
Hydrological	Flood	33	10 (1.8 to 2.6)	30	10 (−0.5 to −1.2)	55	7 (0.4 to 0.5)
	Landslide	6	4 (0.5 to 1.6)	1	15 (−0.5 to −1.1)	9	4 (0.4 to 0.4)
Meteorological	Storm	3	10 (0.5 to 2.6)	3	2 (−0.5 to −0.6)	5	5 (0.0 to 0.4)
	Extreme temperature	1	11 (0.5 to 1.9)	–	–	–	–
Climatological	Drought	7	9 (0.5 to 2.6)	9	9 (−0.5 to −1.1)	3	17 (0.0 to 0.4)
	Wildfire	1	9 (0.5 to 0.9)	–	–	–	–

ENSO, El Niño-Southern Oscillation; ONI, Oceanic Nino Index.

## Discussion

This study highlights the wide range of natural hazards, including climate and environmentally sensitive disease epidemics, that are a major burden affecting millions of people across the Greater Horn of Africa. The open-source databases used in this study were shown to provide a practical basis to initially assess the distributions of natural hazards across this region; however, these databases still feature limitations.

Flood events (hydrological hazards) and cholera (biological hazards) outbreaks were the most common types of natural hazards reported across the Greater Horn of Africa. The most recent annual report for climate state across Africa published by the World Meteorological Organisation (WMO) corroborates these findings, highlighting that ongoing climate change is causing increased extreme flood events, leading to severe loss and damage.^34^ The report states the need for strengthened preparedness against flooding across Africa to prevent loss of life. This may include revised flood responses that rely on increased collaboration between intellectual bodies, governments and project implementers, as highlighted by Turay (2022)^35^ in their systematic review of Africa flood hazard management.

The Horn of African countries have access to climate service platforms such as the IGAD Climate Prediction and Application Centre (ICPAC)^36^ which may help to identify upcoming hazards and risk of extreme climate events such as very heavy rainfall or drought which may be linked to or increase the risk of disease. New tools in development such as the Climate Sensitive Disease Forecasting tool – CLIMSEDIS^28^ may provide additional opportunities for disease control programmes to assess risk based on climate suitability and previous disaster maps.

Our study highlights the importance of understanding data and establishing strategies for cholera to help control long-standing outbreaks and epidemics in line with the Global Task Force on Cholera Control.^37^,^41^ The 2024 weekly epidemiological bulletins highlight the burden and need for control in both South Sudan and Uganda.^42 43^ While there are existing efforts to combat cholera epidemics across Africa, climate change remains a continual and largely unknown threat hindering the progress of such efforts, compounded by the variability in available data and surveillance systems.^44 45^ An assessment of the knowledge, attitude and practice towards cholera across Kenya found gaps in preventative practices.^46^ Therefore, strengthening the health awareness within affected communities through education should be prioritised across the region.

Our study identified potential relationships between natural disasters and epidemics, namely by showing that flooding events may lead to the development of cholera outbreaks.^9 38^ The damage caused by floods may compromise water sanitation infrastructure, leading to contaminated drinking water that can result in the spread of cholera.^38^ Our results are consistent with previous studies, including a spatial-temporal analysis of cholera dynamics across Ethiopia, which found that the spread of cholera was aggravated during floods.^47^ It is likely that a combination of interacting factors is responsible for determining whether an outbreak or epidemic develops and continues. It is also possible that epidemics are under-reported during natural hazards and disasters as epidemiological surveillance may be disrupted during these events. The UNDRR provides a range of risk management strategies which may help address such issues, including, for example, water and sanitation.^48^ The impacts of ongoing climate change are not well understood, resulting in a lack of sufficient multi-hazard early warning systems for epidemics.^34^

We found variability in the number of people affected by the different types of natural hazards. For example, droughts were less frequently reported but affected a larger number of people than recorded by floods. Likewise, a single dengue epidemic in Kenya affected more people than the total number of people affected by all cholera epidemics across the region combined. This exemplifies the need to consider both the frequency of hazard events and the inherent impact that these events have on people when prioritising targeted preparedness. In addition, understanding the extent of previous exposure, levels of immunity and vaccination coverage rates—where applicable—needs to be considered. According to the WHO, the Horn of Africa was recently affected by one of the worst drought periods in recent decades.^49^ The impacts of droughts are expansive and long-lasting, including large-scale population displacements and heightened food insecurity.^49^ Our results provide evidence for the extensive impact that droughts have in this region, especially in Ethiopia, Somalia and Kenya. Altogether, they support the need to integrate climate action into the sustainable development plans of these countries, as suggested by Wright *et al*^50^ in their review of climate change and human health in Africa.

There is also a need to understand how natural hazards and disasters link to climate-related internal displacement and affected disease control programmes.^51^ A recent report by the UNDRR highlighted the significant impact of floods and drought, fuelled by changes fluctuating ENSO across the region with Ethiopia, Kenya and Somalia particularly affected.^52^ In addition, it is critical to consider other societal hazards such as armed conflicts and violence, which significantly increase the risk of social instability, food insecurity, population displacement, limited access to water and sanitation, and potential outbreaks.^53 54^ The use of open-access global databases that focus on these factors such as the Armed Conflict Location & Event Data (ACLED)^55^ and the Internal Displacement Monitoring Centre (iDMC)^56^ should be considered together as a next step. For example, the iDMC’s Global Internal Displacement Database reported 30.06 million conflict and violence-associated internal displacements and 19.82 million disaster-associated internal displacements for the eight Greater Horn of African countries between 2010 and 2024. We need to better understand the complex interactions and challenges, and develop and adapt protocols and strategies in line with global frameworks such as the UNDRR,^48^ WHO Health Emergency and Disaster Risk Framework.^14^

We have shown that open-access databases are useful tools to initially assess the distribution and impacts of natural hazards disasters, and these databases can provide information towards the development of preparedness schemes. However, we found that the data content and reporting consistencies between multiple open-source disaster databases are highly variable. This is clearly demonstrated in the five examples from the different hazards categories. It is also consistent with a study by Cuthbertson *et al*,^57^ who identified substantial variation in disaster reports between the EM-DAT database and an open source hub in Australia. The development of the GLIDE system aims to address this issue.^25^ We further advocate for increased reporting standardisation, including terminology across hazard and disaster databases, potentially with a set of criteria that all organisations may use so comparisons can be made more readily achievable. Consistent terminology would similarly facilitate easier cross-comparisons and make data collation more feasible between these databases.

Further, we acknowledge that the use of open-access data sources may have limitations if they are incomplete, as these sources most likely feature reporting biases and have not recorded all events. Examples of missing data that restricted some of the analysis included (i) incomplete natural hazard start/end dates (missing month—10 records had no start month listed), (ii) missing location information, (iii) missing information on the number of people affected, especially for insect infestations and (iv) missing epidemic ‘name’ (epidemic listed without a given disease) in South Sudan, which is shown in summary figure as an ‘undefined’ disease. This observation is supported with a missing data diagnosis performed by Jones *et al*,^58^ who found a large proportion of missing data for disasters in the EM-DAT database from 1990 to 2020. This highlights the importance of not relying on one database alone and to find practical ways to minimise missing or incomplete data to ensure access to reliable information. More funding is needed for the organisations responsible for these databases to support this critical work and help to strengthen reporting and coordination, especially as climate change continues to present a major global and public health concern.^1^,^3^

While the ENSO phenomenon is a known driver of large-scale climate variability across the Greater Horn of Africa, we found that there was no clear association between one phase of ENSO and the start months of either natural hazards or disasters, though notably drought occurred after a long period in one phase. Despite this, previous work has linked ENSO phases with infectious diseases, such as Moore *et al*,^59^ who found increases in cholera incidence across East Africa during El Niño events. Similarly, Anyamba *et al*^60^ found that disease outbreaks were associated globally with the 2015–2016 El Niño event. Such work suggests that the impact of ENSO patterns on natural hazards and disasters is more important at a lag time of 3–6 months prior to the onset instead of during the month a natural hazard or disaster occurs. Future studies should aim to elucidate further links between ENSO and natural hazards and disasters across the region, taking the length and severity of phases and lag times into account, to maximise the accuracy of climate-based disaster forecasting tools for this region of Africa.

### Conclusion

Natural hazards and related disasters are a major burden across the Greater Horn of Africa, impacting millions of people, and are likely to increase with ongoing climate change. There is an urgent need to improve the understanding of the type and frequency of events, spatial-temporal patterns and their key drivers. This will enable the development of effective disaster risk reduction and disease surveillance and control strategies in line with global policy frameworks across this and other regions in Africa, with an improvement in data reporting consistency, quality and communication and collaboration.

## Supplementary material

10.1136/bmjopen-2025-104998 online supplemental file 1

10.1136/bmjopen-2025-104998 online supplemental file 2

10.1136/bmjopen-2025-104998 online supplemental file 3

10.1136/bmjopen-2025-104998 online supplemental file 4

10.1136/bmjopen-2025-104998 online supplemental file 5

## Data Availability

Data are available in a public, open-access repository. All data relevant to the study are included in the article or uploaded as supplementary information.
