# Supplementary figures and images for: Spatial-temporal analysis of natural hazards and disasters in the Greater Horn of Africa between 2010 and 2024 to inform disaster risk reduction, and surveillance and control strategies for climate and environmentally sensitive diseases
Source: BMJ Open. 2025 Nov 4;15(11):e104998. doi: 10.1136/bmjopen-2025-104998 (PMC12587947; doi:10.1136/bmjopen-2025-104998)

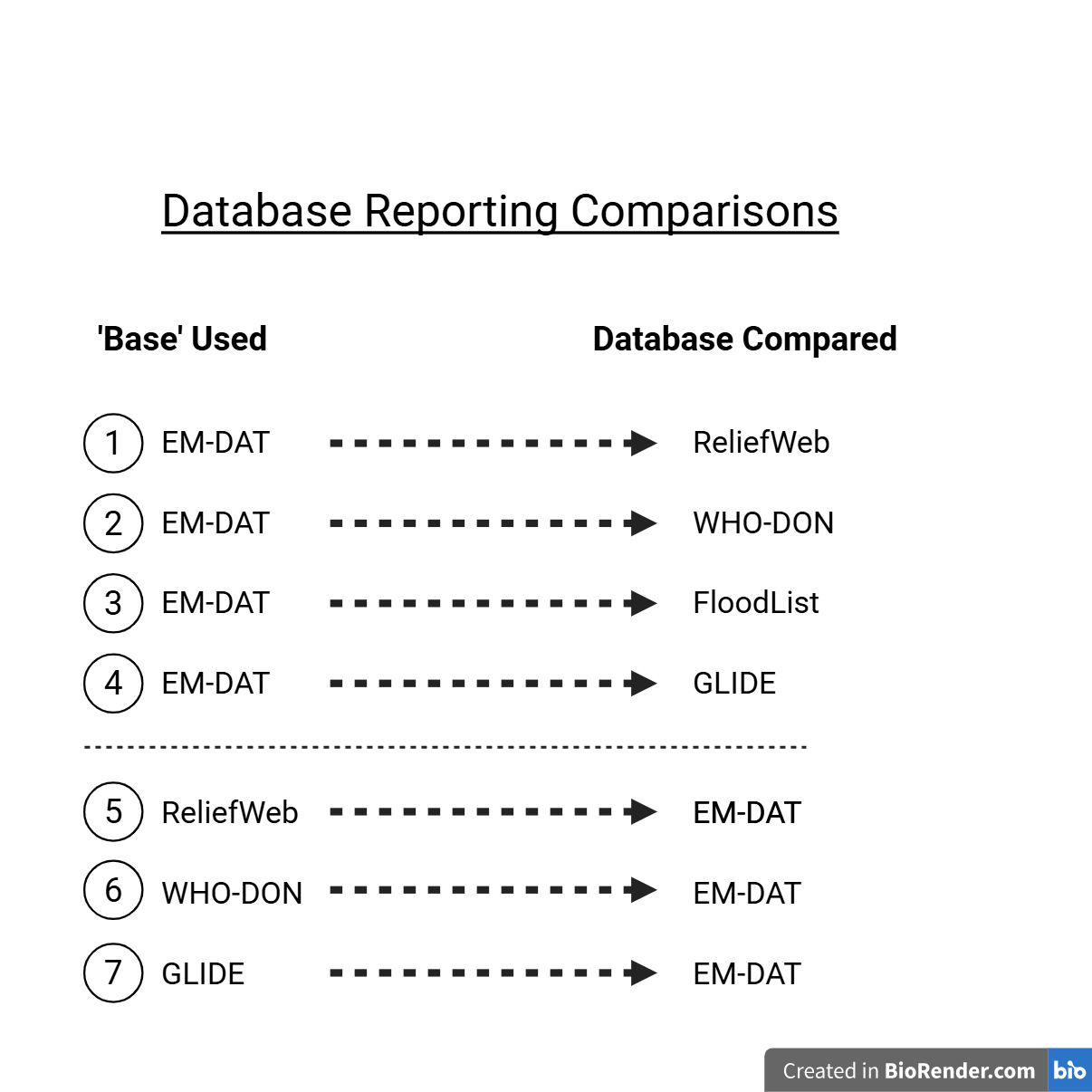

Supplement: online supplemental file 2 [file bmjopen-15-11-s002.png]
