# Supplementary material for: Spatial-temporal analysis of natural hazards and disasters in the Greater Horn of Africa between 2010 and 2024 to inform disaster risk reduction, and surveillance and control strategies for climate and environmentally sensitive diseases
Source: BMJ Open. 2025 Nov 4;15(11):e104998. doi: 10.1136/bmjopen-2025-104998 (PMC12587947; doi:10.1136/bmjopen-2025-104998)
Supplement: online supplemental file 3 [file bmjopen-15-11-s003.docx]

**Supplementary file 3.** Country summaries of A) EM-DAT natural hazards. (B) The total number reported in ReliefWeb, the WHO-DON, FloodList and GLIDE.

A) **Data from EM-DAT**

| **Country** | **Number of Geophysical. Hydrological, Meteorological, and Climatological Hazards** | **Number of Biological Hazards**  **Specifically, Epidemics** | **Number of Insect Infestations** | **Total**  **Number of**  **Natural Hazards or Disasters**  **(% per country)** | |
| --- | --- | --- | --- | --- | --- |
| Djibouti | 5 | No Entries | 1 | 6 (2.6%) | |
| Eritrea | No Entries | No Entries | 1 | 1 (0.4%) | |
| Ethiopia | 26 | 8 | 1 | 35 (15.4%) | |
| Kenya | 36 | 9 | 1 | 46 (20.2%) | |
| Somalia | 33 | 4 | 1 | 38 (16.7%) | |
| South Sudan | 15 | 8 | No Entries | 23 (10.1%) | |
| Sudan | 23 | 7 | 1 | 31 (13.6%) | |
| Uganda | 37 | 10 | 1 | 48 (21.1%) | |
| **Total**  **(% per hazards type)** | **175 (79.2%)** | **46 (20.8%)** | **7 (3.1%)** | **228 (100%)** | |
|  | | | | | |
| B) **Data from ReliefWeb, WHO-DON, FloodList and GLIDE** | | | | | |
| **Country / Database** | **ReliefWeb** | **WHO-DON** | **FloodList** | | **GLIDE** |
| Djibouti | 5 | No Entries | 2 | | 6 |
| Eritrea | 2 | No Entries | No Entries | | 2 |
| Ethiopia | 28 | 4 | 23 | | 46 |
| Kenya | 28 | 12 | 30 | | 51 |
| Somalia | 35 | 7 | 64 | | 38 |
| South Sudan | 24 | 9 | 16 | | 28 |
| Sudan | 25 | 11 | 45 | | 38 |
| Uganda | 23 | 32 | 44 | | 41 |
| **Total** | **170** | **75** | **224** | | **250** |
