# Supplementary material for: Spatial-temporal analysis of natural hazards and disasters in the Greater Horn of Africa between 2010 and 2024 to inform disaster risk reduction, and surveillance and control strategies for climate and environmentally sensitive diseases
Source: BMJ Open. 2025 Nov 4;15(11):e104998. doi: 10.1136/bmjopen-2025-104998 (PMC12587947; doi:10.1136/bmjopen-2025-104998)
Supplement: online supplemental file 4 [file bmjopen-15-11-s004.docx]

**Supplemental file 4.** Summaries of EM-DAT total number of natural hazards of disasters for each country A) per year and B) per month between January 2010 – September 2024

| 1. **Total No. of Natural Hazards or Disasters per Year** | | | | | | | | | | | | | | | |
| --- | --- | --- | --- | --- | --- | --- | --- | --- | --- | --- | --- | --- | --- | --- | --- |
| **Country / Year** | **2010** | **2011** | **2012** | **2013** | **2014** | **2015** | **2016** | **2017** | **2018** | **2019** | **2020** | **2021** | **2022** | **2023** | **2024** |
| Djibouti | 1 | 0 | 0 | 0 | 0 | 0 | 0 | 0 | 1 | 1 | 2 | 0 | 1 | 0 | 0 |
| Eritrea | 0 | 0 | 0 | 0 | 0 | 0 | 0 | 0 | 0 | 0 | 1 | 0 | 0 | 0 | 0 |
| Ethiopia | 3 | 2 | 1 | 4 | 0 | 2 | 2 | 0 | 3 | 5 | 4 | 2 | 3 | 2 | 2 |
| Kenya | 5 | 5 | 1 | 4 | 2 | 3 | 3 | 4 | 3 | 4 | 3 | 2 | 1 | 3 | 3 |
| Somalia | 3 | 0 | 3 | 3 | 2 | 5 | 2 | 1 | 2 | 4 | 3 | 2 | 1 | 6 | 1 |
| South Sudan | 2 | 0 | 2 | 3 | 2 | 1 | 4 | 0 | 0 | 4 | 1 | 2 | 1 | 1 | 0 |
| Sudan | 2 | 1 | 3 | 1 | 1 | 3 | 2 | 2 | 3 | 4 | 3 | 3 | 2 | 0 | 1 |
| Uganda | 2 | 3 | 5 | 4 | 0 | 0 | 3 | 2 | 2 | 8 | 7 | 3 | 6 | 1 | 2 |
| **Total** | **18** | **11** | **15** | **19** | **7** | **14** | **16** | **9** | **14** | **30** | **24** | **14** | **15** | **13** | **9** |
|  | | | | | | | | | | | | | | | |
| 1. **Total No. of Natural Hazards or Disasters per Month** | | | | | | | | | | | | | | | |
| **Country / Month** | **Jan.** | **Feb.** | **Mar.** | **Apr.** | **May** | **Jun.** | **Jul.** | **Aug.** | **Sep.** | **Oct.** | **Nov.** | **Dec.** | **Undefined Start Month*** | | |
| Djibouti | 0 | 1 | 0 | 1 | 1 | 1 | 0 | 0 | 0 | 0 | 2 | 0 | 0 | | |
| Eritrea | 0 | 1 | 0 | 0 | 0 | 0 | 0 | 0 | 0 | 0 | 0 | 0 | 0 | | |
| Ethiopia | 2 | 0 | 2 | 5 | 6 | 1 | 3 | 2 | 3 | 5 | 3 | 1 | 2 | | |
| Kenya | 7 | 1 | 8 | 10 | 3 | 1 | 0 | 2 | 0 | 4 | 3 | 6 | 1 | | |
| Somalia | 3 | 2 | 4 | 6 | 4 | 1 | 1 | 2 | 2 | 4 | 6 | 3 | 1 | | |
| South Sudan | 2 | 2 | 0 | 2 | 3 | 1 | 3 | 5 | 1 | 3 | 1 | 0 | 1 | | |
| Sudan | 0 | 1 | 0 | 0 | 1 | 7 | 6 | 8 | 3 | 0 | 0 | 0 | 5 | | |
| Uganda | 4 | 3 | 1 | 5 | 7 | 3 | 2 | 7 | 6 | 4 | 3 | 3 | 0 | | |
| **Total** | **18** | **11** | **15** | **29** | **25** | **15** | **15** | **26** | **15** | **20** | **18** | **13** | **10** | | |

**Note.** * Natural hazards or disasters without a defined starting month are included in the ‘Undefined Start Month’ column.
