# Supplementary material for: Spatial-temporal analysis of natural hazards and disasters in the Greater Horn of Africa between 2010 and 2024 to inform disaster risk reduction, and surveillance and control strategies for climate and environmentally sensitive diseases
Source: BMJ Open. 2025 Nov 4;15(11):e104998. doi: 10.1136/bmjopen-2025-104998 (PMC12587947; doi:10.1136/bmjopen-2025-104998)
Supplement: online supplemental file 5 [file bmjopen-15-11-s005.docx]

**Supplementary file 8** Tabulated summaries of ENSO phases at the onset months of EM-DAT January 2010- September 2024 for A) natural hazards excluding biological and B) epidemics, per country.

| 1. **ENSO Phase During Natural Disaster Onset Month** | | | |
| --- | --- | --- | --- |
| **Country** | Phase According to ONI | | |
|  | **El Niño** | **La Niña** | **Neutral** |
| Djibouti | 2 | 2 | 2 |
| Eritrea | 1 | 0 | 0 |
| Ethiopia | 8 | 6 | 11 |
| Kenya | 13 | 9 | 14 |
| Somalia | 15 | 5 | 13 |
| Sudan | 5 | 4 | 13 |
| South Sudan | 2 | 5 | 7 |
| Uganda | 11 | 14 | 12 |
| **Total** | **57** | **45** | **72** |
|  | | | |
| 1. **ENSO Phase During Epidemic Onset Month** | | | |
| **Country** | Phase According to ONI | | |
|  | **El Niño** | **La Niña** | **Neutral** |
| Djibouti | 0 | 0 | 0 |
| Eritrea | 0 | 0 | 0 |
| Ethiopia | 4 | 1 | 3 |
| Kenya | 3 | 5 | 1 |
| Somalia | 2 | 1 | 1 |
| Sudan | 0 | 1 | 3 |
| South Sudan | 3 | 1 | 4 |
| Uganda | 0 | 4 | 6 |
| **Total** | **12** | **13** | **18** |
