# Supplementary material for: Spatial-temporal analysis of natural hazards and disasters in the Greater Horn of Africa between 2010 and 2024 to inform disaster risk reduction, and surveillance and control strategies for climate and environmentally sensitive diseases
Source: BMJ Open. 2025 Nov 4;15(11):e104998. doi: 10.1136/bmjopen-2025-104998 (PMC12587947; doi:10.1136/bmjopen-2025-104998)
Supplement: Supplementary file 6 [file bmjopen-2025-104998.reviewer_comments.pdf]

PEER REVIEW HISTORY

BMJ Open publishes all reviews undertaken for accepted manuscripts. Reviewers are asked to complete a checklist review form and are provided with free text boxes to elaborate on their assessment. These free text comments are reproduced below.

ARTICLE DETAILS

Title (Provisional)

Spatial-temporal analysis of natural hazards and disasters in the Greater Horn of Africa between 2010 and 2024 to inform disaster risk reduction, and surveillance and control strategies for climate- and environmentally- sensitive diseases.

Authors

Norris, Luke N; Lemin, Morgan; Kelly-Hope, Louise

VERSION 1 - REVIEW

Reviewer **1**

Name **Egawa, Shinichi**

Affiliation **Tohoku University, International Cooperation for Disaster Medicine Lab.,International Research Institute of Disaster Science (IRIDeS)**

Date **07-Jul-2025**

COI **None**

General comment

This is a publicly available database analysis on the disasters in the Greater Horn of Africa. First, I would like to clarify the terminology used in this manuscript. Essentially, the authors should adhere to the UNDRR terminology. The EM-DAT is well-known, but a single disaster ID system (GLIDE system) was established, featuring a wide contribution and collaboration of experts and organizations in disaster statistics, including EM-DAT. Thus, I strongly recommend sticking to the GLIDE system rather than EM-DAT.

Secondly, as the title of this manuscript indicates, the sequential occurrence of epidemics after natural hazard events is often underreported. Thus, the causal relationship cannot be elucidated solely from this database analysis. However, this study suggests that the independent epidemics may arise several months after the hazards attack in the same are (districts). In that sense, I doubt the validity of restricting the hazard type to climate-related ones. WHO is recommending an all-hazard approach. And the GLIDE system includes societal hazards such as armed conflicts and violence, which significantly increase social instability, food insecurity, water and sanitation, and people’s mental health. Without mentioning the root causes of this background, the notion of this paper misleads the readers.

Thirdly, the terminology should be clarified. Follow the UNDRR terminology (https://www.undrr.org/drr-glossary/terminology). A fundamental understanding of disaster risk is needed. Disaster is a function of Hazard, Exposure, Vulnerability, and Coping Capacity, as used in the public disaster risk database, the INFORM Risk Index (https://drmkc.jrc.ec.europa.eu/inform-index), which I recommend incorporating into this manuscript to evaluate the disaster risk indices in the target countries.

The following points should be corrected:

Line 3: Avoid using "natural disasters." There are "natural hazards," but the disaster is a consequence of human factors, including exposures, cascading events, vulnerabilities, and coping capacities. Use "natural hazards" or "disasters" according to the context.

Line 43 and throughout the manuscript: Use "natural hazards" instead of "natural disasters." Epidemics are also a type of disaster due to natural hazards because the pathogens are natural. See WHO's Health Emergency and Disaster Risk Reduction Framework (https://iris.who.int/bitstream/handle/10665/326106/9789241516181-eng.pdf).

Line 45: Distinguish hazards and disasters.

Line 83: Use "hazards" instead of "disasters."

Line 91: When you substitute "natural disasters" with "natural hazards," extreme climate events are included in the natural hazards. See the WHO classification of hazards (Appendix 1) in the H-EDRM framework carefully and modify the expressions. There are environmental hazards, which cannot be classified as "natural" or "human-induced" hazards, as you point out the effect of both consequences. Consider "all hazards" approach.

Line 98: EM-DAT is now contributing to the GLIDEnumber2.0 database which might be a larger database including the disaster IDs, which is different from the serial number in EMDAT. During 2010-2024, there are overall 252 disasters in these countries in GLIDE. The hazard types include 11 complex emergencies, 24 droughts, 78 epidemics, 2 fires, 14 flash floods, 83 floods, two insect infestations, 4 landslides, one mud slide, four tropical cyclones, one violent wind, and 28 others. All of them were verified and identified by the cooperation with EMDAT. GLIDE is including 38 complex and other disasters mainly due to human-induced hazards underscoring the social vulnerabilities in this region. Consider your database.

Line 104: What does the "disaster-related mitigation" mean? Refer to the UNDRR terminologies. I recommend using disaster risk reduction (DRR) in this context.

Line 107: I agree that cascading events after natural hazards are now the topic to discuss. However, epidemics can even follow human-induced events. A typical example is the cholera outbreak in Haiti after the 2010 Earthquake. But the outbreak was confirmed as a consequence of the relief aid 10 years later. Social instability may have contributed to the vulnerabilities, including the WASH situation. Thus, we must be cautious about the causal relationship, and the research question "subsequent epidemics" should be carefully justified,

explaining why this question was chosen. Because you have decided on the countries based on the CLIMSEDIS, climatic hazards, and any health impacts (for example, malnutrition and hyperthermia), these can be analyzed. Please rationalize why you chose epidemics.

Line 145: If you are including these hazards, epidemics, and infestations collectively as "natural hazards," the inclusiveness is reversed. Disaster includes hazards, but hazards do not include disaster. Classify the types of hazard according to the WHO H-EDRM classification, for example. Then, the epidemics are a consequence of the biological hazards, which include insect infestations. The GLIDE system includes the outbreak of hazard, SIRS-CoV-2, the disaster type is epidemics (COVID-19). Thus, listing epidemics as a type of hazard is misleading.

Line 151: Reconsider the expression.

Line 157: Use "hazard types" instead of "natural disasters, epidemics, and infestations."

Line 158: Use "disasters" instead of "natural disasters and epidemics."

Line 158: Remove the last "natural."

Line 165: I strongly recommend using the disaster IDs listed in GLIDE2.0. As you mentioned, it is not easy for anyone to identify and collate the overlap of disastrous events from various sources by name, date, and type, because many non-medical experts describe disasters from multiple aspects. The GLIDE system is designed to avoid such confusion (https://glidenumber.net/glide/public/about.jsp).

Line 185: I strongly insist that the Supplementary File 1 should include the GLIDE numbers. This reduces your workload and increases the reliability of this study in line with international initiatives.

Line 197: Clearly distinguish hazards and disasters. Disasters due to natural hazards include epidemics such as the Ebola pandemic and the COVID-19 pandemic. The terminology is crucial for making this study clear. If you want to evaluate an infectious disease outbreak following natural hazards, describe it accordingly. However, as mentioned earlier, the health consequences of natural hazards are not limited to infectious disease outbreaks.

Line 200: If you include geophysical hazards, they are not climate-related. If you are focusing on the hydrometeorological hazards, describe them. There might be a climatic impact on the insect infestations. On the other hand, many might doubt the causal relationship when COVID-19 occurred in the region within 3 months after an extreme weather event, that this is due to the climate. Reconsider the expression.

Lines 205 and 210: The hazard types should be restricted to the hydrometeorological ones.

Line 209: Remove the brackets around NOAA.

Line 229: Use "disasters" instead of "natural hazards."

Line 230: Use hydrometeorological hazards if you focus on climate-related disease outbreaks.

Line 233: The summary of the number of disasters will change if you use GLIDE.

Lines 240-244: Use "hazards" where appropriate.

Line 272: I don't think Table 1 is necessary for the "spatial and temporal analysis" of natural hazards and disease outbreaks.

Line 289: Eritrea was affected by the DR-2022-000239-ERI (drought), and EP-2020-000012-ERI (COVID-19).

Line 294: The data is incomplete without including COVID-19 during the targeted period. When you say "spatial and temporal" analysis, mapping, and the onset-ending details are necessary to consider the causal relationships rather than the ranking of countries.

Line 302: Use "hazard type" instead of "natural disaster."

Line 312: I don't think the comparison between the public databases and consistency analyses is necessary if you use the GLIDE system.

Line 340: Using the GLIDE system will enable you to avoid these comparisons.

Line 350-357: Use "hazards" where appropriate. Since you did not include the COVID-19 pandemic data for each country, the information may be incomplete. Another significant limitation is that infectious disease outbreaks immediately following the hazard event were not reported as separate epidemics. Thus, you have detected the epidemic events within 3 months in the natural hazard-affected area. I also want to raise a concern about the societal hazard in these countries (there are tens of conflicts and complex disasters in the GLIDE system in this region), which will make vulnerabilities within the affected community, including food insecurity, water hygiene, and lower accessibility to the healthcare system. I think this section of the results is the most relevant to the notion of this manuscript. Enrich the content and refine the results.

Line 379: The average length of each ENSO phase and its relation to climate change should be summarized here. Otherwise, readers cannot grasp the validity of the results. Was the neutral phase the longest? At which phase do floods, storms, torrential rains, high temperatures, and wildfires occur? The supplementary Table is not helpful in seeing the relationships between ENSO phase and hydrometeorological hazards.

Line 386: The terminology is misleading. There is no "natural disasters." Natural hazards include infectious pathogens. Correct.

Line 387: When discussing the vulnerability of people living in this area, the public database and the INFORM risk index are beneficial for evaluating the risks. Without considering the societal hazards and complex disasters, it is not possible to discuss the vulnerabilities of people.

Line 389: After introducing the GLIDE system, the limitation of the database could be decreased (I do agree that the database is still far from complete).

Line 402: CLIMSEDIS was the reason for determining the target countries of the Greater Horn. Is the data from CLIMSEDIS available like WHO-DON?

Line 423: Use "disasters" instead of "natural disasters."

Line 426: There could be multiple aspects of disaster risk reduction. Consider the terminology "disaster risk reduction (DRR)" as the central working strategy for this study. Disasters are the consequences of disaster risks defined by the function of Hazard & Exposure, Vulnerability, and Coping Capacity. Thus, many measures can be taken to reduce disaster risks during ordinary times (anticipating the next disaster). Once the event occurs (onset), response, recovery, and reconstruction are necessary. If people are living in a low-lying area prone to flooding, relocating to an elevated location may be possible. The failure of DRR will create new vulnerabilities.

Line 433: "targeted preparedness against disasters" can be substituted by DRR. Reconsider the expression.

Line 444 paragraph: This discussion is half solved by the GLIDE system. Reorganize the discussion.

Line 446: Consider using DRR instead of "natural hazard preparedness schemes."

Line 475: In Table 2, the epidemics are occurring after flood events, which is understandable due to the hygiene problems. Such a discussion and the DRR measures should be discussed.

Line 480: Reorganize the terminologies.

Line 483: I suppose that the key drivers require a more transdisciplinary approach as illustrated in the WHO Health EDRM framework. The health sector should cooperate with other sectors not only for the DRR, but also for social development and governments. Reconsider the expression.

**Reviewer**	**2**
**Name**	**Kelson, Zoe**
**Affiliation**	**University of Exeter, Mathematics**
**Date**	**17-Aug-2025**
**COI**	**None**

This cross-sectional study of historical data aimed to determine the spatial-temporal patterns of natural disasters and disease outbreaks across eight Horn of African countries and compare the reporting consistencies across multiple open-access databases.

Reviewer comments:

"Specifically, we aimed to (1) summarise the spatial-temporal distribution of disasters and their impacts (2) examine the relationship between natural disasters and subsequent epidemics (3) compare the reporting consistencies across multiple open-source databases and (4) determine if phases of ENSO are linked to disaster onset. "

Can the authors please make it clear in the Title and objectives of this study that a descriptive analysis only has been reported?

Can the authors please consider conducting statistical models to assess spatio-temporal trends?

"We focused our study on eight countries within the Greater Horn of Africa: Djibouti, Eritrea, Ethiopia, Kenya, Somalia, Sudan, South Sudan and Uganda"

Can the authors please comment on whether these cohorts are representative for generalisability of the study outcomes to the Great Horn of Africa?

"Data from the Emergency Events Database (EM-DAT) between 2010 – 2024 was used as a reference database, and cross-referenced with data from Reliefweb, FloodList and the WHO Disease Outbreak News"

and

"The data sources and distinctions are listed here. EM-DAT: Records 'Natural Disasters,' 'Epidemics' and 'Infestations.' Data downloadable from website. ReliefWeb: Records 'Natural Disasters,' 'Epidemics' and 'Infestations.' Data listed on website. WHO-DON: Records 'Disease Outbreaks'. Data listed on website. FloodList: Records 'Flood Events.' Described through news reports and articles. Data listed on website."

Can the authors please further comment on the completeness and coverage of this data?

"For the period of January 2010 – September 2024, disaster data were extracted from EM-DAT and compiled into a single datasheet, including information on the disaster type, subtype, start date, end date, country, location, event name and the total number of people affected listed"

Can the authors please confirm if there was any missing data?

"To compare the reporting consistencies across multiple open-access data sources, we extracted data for the same period from three additional open-access disaster databases / resources that report on natural disasters and epidemics or outbreaks, ReliefWeb, the WHO-DON and FloodList. "

and

"As a singular disaster report in one database could be considered the same as multiple data entries within another database, we calculated the proportion of disasters for each 'reference database' that could be considered the same event across all five database comparisons carried out. "

Can a sensitivity analysis on this approach, excluding possible duplicate entry across databases, please be considered?

"To examine the relationship between natural disasters and subsequent epidemics, we examined the EM-DAT data to identify natural disasters that had occurred within 3 months prior to an epidemic in the same reported location (district)."

Can a sensitivity analysis on the choice of a 3 month window to assess this relationship please be considered?

Can the authors please add a Statistical Analysis section to describe the methods they used to analyse the data?

"Figure 1. Summaries of EM-DAT January 2010 – September 2024 on the distribution of A) natural disasters subtype and B) epidemic subtype per country. "

and

"Figure 2. Summary of EM-DAT data January 2010 – September 2024. Graphs showing (A) the total number of people affected by each natural disaster and (B) the total number of people affected by each epidemic. "

Can distributions please also be shown and analysed over time?

Can the Tables please be reformatted for improved readability (currently they overlap pages and are difficult to interpret)?

"Open-access disaster database sources vary in the type of information they collect and have several data gaps, including limited georeferenced information, which restricts comparability and opportunities for detailed mapping and analysis. " [Strengths and Limitations of this data]

and

"We acknowledge that the use of open access data sources may have limitations if they are 221 incomplete as these sources most likely feature reporting biases and have not recorded all events. 222 We also acknowledge that our focus on a few selected data sources may cause unforeseen biases." [Data Limitations]

The authors suitably acknowledge the potential biases in the data.

Can the study limitations please be further addressed in the Discussion section of the article?

Thanks for providing a copy of the STROBE checklist.

VERSION 1 - AUTHOR RESPONSE

Reviewer: 1
Dr. Shinichi Egawa, Tohoku University
Comments to the Author:
General comment
This is a publicly available database analysis on the disasters in the Greater Horn of Africa. First, I would like to clarify the terminology used in this manuscript. Essentially, the authors should adhere to the UNDRR terminology. The EM-DAT is well-known, but a single disaster ID system (GLIDE system) was established, featuring a wide contribution and collaboration of experts and organizations in disaster statistics, including EM-DAT. Thus, I strongly recommend sticking to the GLIDE system rather than EM-DAT.

Response: Thank you for your comprehensive expert review. We have responded all comments which are detailed below.

We have included UNDRR information in the introduction and have clarified the differences in the way organisation refer to natural hazards and disasters according to their focus and directive. We have included a table showing how the UNDRR and EM-DAT terminologies align. We have revised the terminology throughout, in addition to your specific suggestions.

We have included reference to GLIDE as an additional database with data added to the main natural hazard file. We have kept the EM-DAT as the main reference database and showed the comparisons between four additional databases, including GLIDE, which we found also did not align or capture all hazard events. In addition to the addition reference in the databases, we have added in examples of how the content of the various databases vary and all provide different aspects of the event.

Secondly, as the title of this manuscript indicates, the sequential occurrence of epidemics after natural hazard events is often underreported. Thus, the causal relationship cannot be elucidated solely from this database analysis. However, this study suggests that the independent epidemics may arise several months after the hazards attack in the same are (districts). In that sense, I doubt the validity of restricting the hazard type to climate-related ones. WHO is recommending an all-hazard approach. And the GLIDE system includes societal hazards such as armed conflicts and violence, which significantly increase social instability, food insecurity, water and sanitation, and people’s mental health. Without mentioning the root causes of this background, the notion of this paper misleads the readers.

Response: Title has been modified as per reviewer 1 comments, and to inform of the period and focus on environmentally sensitive diseases.

We have included more clarification/rational in the methods for the reason why we are focusing on the natural hazards and disasters [i.e. our research project]. We appreciate there are many complex and interacting factors, however it is beyond the scope of this paper to include societal related hazards, including conflict. We acknowledge that databases such as GLIDE are now starting to include such data, however these are still relatively limited and still would require extensive cross-referencing with key databases such as ACLED and ICDM which we have noted in the discussion as a next step and provided some context in terms of the numbers of people affected.

Thirdly, the terminology should be clarified. Follow the UNDRR terminology (https://www.undrr.org/drr-glossary/terminology). A fundamental understanding of disaster risk is needed. Disaster is a function of Hazard, Exposure, Vulnerability, and Coping Capacity, as used in the public disaster risk database, the INFORM Risk Index (https://drmkc.jrc.ec.europa.eu/inform-index), which I recommend incorporating into this manuscript to evaluate the disaster risk indices in the target countries.

Response: We have added more on the UNDRR definitions and terminology in the introduction with reference to the latest materials. We have adjusted the methods to include a table to compare the UNDRR and EM-DAT terminology. https://www.undrr.org/drr-glossary/terminology We have also noted how the UNDRR aligns with and supports global frameworks such as the WHO health EDRM and Sendai Framework for examples.

The following points should be corrected:
# Line 3: Avoid using "natural disasters." There are "natural hazards," but the disaster is a consequence of human factors, including exposures, cascading events, vulnerabilities, and coping capacities. Use "natural hazards" or "disasters" according to the context.

Response; We have updated the terminology throughout the whole document.

# Line 43 and throughout the manuscript: Use "natural hazards" instead of "natural disasters." Epidemics are also a type of disaster due to natural hazards because the pathogens are natural. See WHO's Health Emergency and Disaster Risk Reduction Framework (https://iris.who.int/bitstream/handle/10665/326106/9789241516181-eng.pdf).

Response; We have updated the terminology throughout the whole document. We have also include reference to this source in the introduction.

# Line 45: Distinguish hazards and disasters. Done

# Line 83: Use "hazards" instead of "disasters." Done

# Line 91: When you substitute "natural disasters" with "natural hazards," extreme climate events are included in the natural hazards. See the WHO classification of hazards (Appendix 1) in the H-EDRM framework carefully and modify the expressions. There are environmental hazards, which cannot be classified as "natural" or "human-induced" hazards, as you point out the effect of both consequences. Consider "all hazards" approach. Done

Response to five points: Done

# Line 98: EM-DAT is now contributing to the GLIDEnumber2.0 database which might be a larger database including the disaster IDs, which is different from the serial number in EMDAT. During 2010-2024, there are overall 252 disasters in these countries in GLIDE. The hazard types include 11 complex emergencies, 24 droughts, 78 epidemics, 2 fires, 14 flash floods, 83 floods, two insect infestations, 4 landslides, one mud slide, four tropical cyclones, one violent wind, and 28 others. All of them were verified and identified by the cooperation with EMDAT. GLIDE is including 38 complex and other disasters mainly due to human-induced hazards underscoring the social vulnerabilities in this region. Consider your database.

Response: A noted above we have now included GLIDE as a comparative database. We found that there is some discrepancies. As noted in this reports, there is improved data cross-referencing, however, more efforts are still required to link all events. https://files.emdat.be/2025/08/CredCrunch79.pdf

# Line 104: What does the "disaster-related mitigation" mean? Refer to the UNDRR terminologies. I recommend using disaster risk reduction (DRR) in this context.

Response: We have deleted this as it is misleading and the section has been revised overall.

# Line 107: I agree that cascading events after natural hazards are now the topic to discuss. However, epidemics can even follow human-induced events. A typical example is the cholera outbreak in Haiti after the 2010 Earthquake. But the outbreak was confirmed as a consequence of the relief aid 10 years later. Social instability may have contributed to the vulnerabilities, including the WASH situation. Thus, we must be cautious about the causal relationship, and the research question "subsequent epidemics" should be carefully justified, explaining

why this question was chosen. Because you have decided on the countries based on the CLIMSEDIS, climatic hazards, and any health impacts (for example, malnutrition and hyperthermia), these can be analyzed. Please rationalize why you chose epidemics.

Response: We have provided more rational in the methods as to why we focus on the natural hazards and disasters.

# Line 145: If you are including these hazards, epidemics, and infestations collectively as "natural hazards," the inclusiveness is reversed. Disaster includes hazards, but hazards do not include disaster. Classify the types of hazard according to the WHO H-EDRM classification, for example. Then, the epidemics are a consequence of the biological hazards, which include insect infestations. The GLIDE system includes the outbreak of hazard, SIRS-CoV-2, the disaster type is epidemics (COVID-19). Thus, listing epidemics as a type of hazard is misleading.

Response: As noted above we have revised the terminologies we use which is based on the EM-DAT classifications, which are well aligned with the UNDRR terminology as shown in the new Table 1.

# Line 151: Reconsider the expression.
Response: This has been revised.

# Line 157: Use "hazard types" instead of "natural disasters, epidemics, and infestations."

Response: Done

# Line 158: Use "disasters" instead of "natural disasters and epidemics."

Response: Done

# Line 158: Remove the last "natural."

Response: Done

# Line 165: I strongly recommend using the disaster IDs listed in GLIDE2.0. As you mentioned, it is not easy for anyone to identify and collate the overlap of disastrous events from various sources by name, date, and type, because many non-medical experts describe disasters from multiple aspects. The GLIDE system is designed to

avoid such confusion (https://glidenumber.net/glide/public/about.jsp).

Response: We have added in the GLIDE numbers to help provide linkage between the information across the databases. We have also included a new section on the content of each database in five examples, which highlight the different aspects and information they provide, all of which have value.

# Line 185: I strongly insist that the Supplementary File 1 should include the GLIDE numbers. This reduces your workload and increases the reliability of this study in line with international initiatives.

Response: We have included the GLIDE data in the main databased and highlighted the ones that matched and were unable to be found. We also have added an additional supplementary file with all GLIDE data for the time.

# Line 197: Clearly distinguish hazards and disasters. Disasters due to natural hazards include epidemics such as the Ebola pandemic and the COVID-19 pandemic. The terminology is crucial for making this study clear. If you want to evaluate an infectious disease outbreak following natural hazards, describe it accordingly. However, as mentioned earlier, the health consequences of natural hazards are not limited to infectious disease outbreaks.

Response: We have rephrased this comparative work in line with the changed wording in the objectives. The terminology throughout the paper has been updated. We focus on infectious diseases for the purpose of the Climsedis project as noted above and in the methods section.

# Line 200: If you include geophysical hazards, they are not climate-related. If you are focusing on the hydrometeorological hazards, describe them. There might be a climatic impact on the insect infestations. On the other hand, many might doubt the causal relationship when COVID-19 occurred in the region within 3 months after an extreme weather event, that this is due to the climate. Reconsider the expression.

Response: This has been rephrased ad reference to climate removed.

# Lines 205 and 210: The hazard types should be restricted to the hydrometeorological ones.

Response: We have included the review of all natural hazards and disasters as the ENSO has the potential to influence all, and not just hydrometeorological ones.

# Line 209: Remove the brackets around NOAA.

Response: Done

# Line 229: Use "disasters" instead of "natural hazards."

Response: Done

# Line 230: Use hydrometeorological hazards if you focus on climate-related disease outbreaks.

Response. We have removed reference to climate specifically as they can be environmentally sensitive which takes a wider range of influencing factors into account.

# Line 233: The summary of the number of disasters will change if you use GLIDE.

Response: As noted above, we used EM-DAT as our primary reference database and have highlighted the linkages with GLIDE and other databases, including the data that matched and where there are discrepancies.

# Lines 240-244: Use "hazards" where appropriate.

Response: Done

# Line 272: I don't think Table 1 is necessary for the "spatial and temporal analysis" of natural hazards and disease outbreaks.

Response: We have kept in as it is informative for reader to see the data and numbers for the years and month for each of the eight countries.

# Line 289: Eritrea was affected by the DR-2022-000239-ERI (drought), and EP-2020-000012-ERI (COVID-19).

Response: This was captured in the GLIDE download. We highlighted that no COVID-19 epidemics were listed in EM-DAT data. While EM-DAT did not list these events, it did list an insect infestation affecting Eritrea that was not could be found in GLIDE.

# Line 294: The data is incomplete without including COVID-19 during the targeted period. When you say "spatial and temporal" analysis, mapping, and the onset-ending details are necessary to consider the causal relationships rather than the ranking of countries.

Response: We focused only what is reported in the EM-DAT database and appreciate that other diseases will not be included in this database, but to include all the possible diseases from other databases in beyond the scope of this paper..

# Line 302: Use "hazard type" instead of "natural disaster."

Response: Done

# Line 312: I don't think the comparison between the public databases and consistency analyses is necessary if you use the GLIDE system.

Response: We acknowledge the GLIDE system, however, still think it is important to highlight the inconsistencies and as noted above we did not find that GLIDE captured all events.

# Line 340: Using the GLIDE system will enable you to avoid these comparisons.

Response: We have added reference to this in the discussion.

# Line 350-357: Use "hazards" where appropriate. Since you did not include the COVID-19 pandemic data for each country, the information may be incomplete. Another significant limitation is that infectious disease outbreaks immediately following the hazard event were not reported as separate epidemics. Thus, you have detected the epidemic events within 3 months in the natural hazard-affected area. I also want to raise a concern about the societal hazard in these countries (there are tens of conflicts and complex disasters in the GLIDE system in this region), which will make vulnerabilities within the affected community, including food insecurity, water hygiene, and lower accessibility to the healthcare system. I think this section of the results is the most relevant to the notion of this manuscript. Enrich the content and refine the results.

Response: We used EM-DAT as a base and only reported on the data within the database. It is beyond the scope of this paper to include all databases on diseases..

# Line 379: The average length of each ENSO phase and its relation to climate change should be summarized here. Otherwise, readers cannot grasp the validity of the results. Was the neutral phase the longest? At which phase do floods, storms, torrential rains, high temperatures, and wildfires occur? The supplementary Table is not helpful in seeing the relationships between ENSO phase and hydrometeorological hazards.

Response: We have included more information on the length of the ENSO phase and the ONI range, which are included in the paper to be more informative.

# Line 386: The terminology is misleading. There is no "natural disasters." Natural hazards include infectious pathogens. Correct.

Response: We have updated the terminology to address this.

# Line 387: When discussing the vulnerability of people living in this area, the public database and the INFORM risk index are beneficial for evaluating the risks. Without considering the societal hazards and complex disasters, it is not possible to discuss the vulnerabilities of people.

Response: We have added more in the discussion on the societal hazards and complex interacting challenges that people face. It is beyond the scope of this paper to include analysis of the INFORM risk index.

# Line 389: After introducing the GLIDE system, the limitation of the database could be decreased (I do agree that the database is still far from complete).

Response: We have included links to the GLIDE data and highlighted the missing hazards, which also in turn highlights the inconsistencies between the databases and one of the objectives of the paper.

# Line 402: CLIMSEDIS was the reason for determining the target countries of the Greater Horn. Is the data from CLIMSEDIS available like WHO-DON?

Response: The Greater Horn of Africa was chosen as it is the study site for the Wellcome Trust funded project. We have clarified this in the paper.

# Line 423: Use "disasters" instead of "natural disasters."

Response: Done

# Line 426: There could be multiple aspects of disaster risk reduction. Consider the terminology "disaster risk reduction (DRR)" as the central working strategy for this study. Disasters are the consequences of disaster risks defined by the function of Hazard & Exposure, Vulnerability, and Coping Capacity. Thus, many measures can be taken to reduce disaster risks during ordinary times (anticipating the next disaster). Once the event occurs (onset), response, recovery, and reconstruction are necessary. If people are living in a low-lying area prone to flooding, relocating to an elevated location may be possible. The failure of DRR will create new vulnerabilities.

Response: As noted above, we have used the EM-DAT terminology which align with the UNDRR and provided a new table to show this.

# Line 433: "targeted preparedness against disasters" can be substituted by DRR. Reconsider the expression.

Response: Done

# Line 444 paragraph: This discussion is half solved by the GLIDE system. Reorganize the discussion.

Response: We added to the discussion to highlight some key aspects further, including the data inconsistencies, content variability and societal hazards.

# Line 446: Consider using DRR instead of "natural hazard preparedness schemes."

Response: Done

# Line 475: In Table 2, the epidemics are occurring after flood events, which is understandable due to the hygiene problems. Such a discussion and the DRR measures should be discussed.

Response: This is a helpful point and we have added in reference the to the UNDRR risk management strategies, specifically water and sanitation.

# Line 480: Reorganize the terminologies.

Response: Done

# Line 483: I suppose that the key drivers require a more transdisciplinary approach as illustrated in the WHO Health EDRM framework. The health sector should cooperate with other sectors not only for the DRR, but also for social development and governments. Reconsider the expression.

Response: We have included reference to WHO Health EDRM in the introduction and discussion.

Reviewer: 2

Prof. Zoe Kelson, University of Exeter
Comments to the Author:
This cross-sectional study of historical data aimed to determine the spatial-temporal patterns of natural disasters and disease outbreaks across eight Horn of African countries and compare the reporting consistencies across multiple open-access databases.

Reviewer comments:

"Specifically, we aimed to (1) summarise the spatial-temporal distribution of disasters and their impacts (2) examine the relationship between natural disasters and subsequent epidemics (3) compare the reporting consistencies across multiple open-source databases and (4) determine if phases of ENSO are linked to disaster onset. "
Can the authors please make it clear in the Title and objectives of this study that a descriptive analysis only has been reported?

Response: We have updated the title

Can the authors please consider conducting statistical models to assess spatio-temporal trends?

Response: Statistical models on this data were not possible, however we have included statistical analysis on the differences between spatial and temporal patterns. The descriptions of each analysis have been added in the relevant sections of the methods.

"We focused our study on eight countries within the Greater Horn of Africa: Djibouti, Eritrea, Ethiopia, Kenya, Somalia, Sudan, South Sudan and Uganda"

Can the authors please comment on whether these cohorts are representative for generalisability of the study outcomes to the Great Horn of Africa?

Response: The countries represent the region well and we have noted that this approach of using hazard and disaster data could be applied to other regions in Africa and globally.

"Data from the Emergency Events Database (EM-DAT) between 2010 – 2024 was used as a reference database, and cross-referenced with data from Reliefweb, FloodList and the WHO Disease Outbreak News"
and
"The data sources and distinctions are listed here. EM-DAT: Records 'Natural Disasters,' 'Epidemics' and 'Infestations.' Data downloadable from website. ReliefWeb: Records 'Natural Disasters,' 'Epidemics' and 'Infestations.' Data listed on website. WHO-DON: Records 'Disease Outbreaks'. Data listed on website. FloodList: Records 'Flood Events.' Described through news reports and articles. Data listed on website."
Can the authors please further comment on the completeness and coverage of this data?
"For the period of January 2010 – September 2024, disaster data were extracted from EM-DAT and compiled into a single datasheet, including information on the disaster type, subtype, start date, end date, country, location, event name and the total number of people affected listed"
Can the authors please confirm if there was any missing data?

Response: Can confirm that data was missing in our primary database EM-DAT. These are also evident in the database provided – supplemental file 2.

Examples of missing data that restricted some of the analysis included:

- *Incomplete start/end dates (missing month – 10 disasters had no start month listed)*
- *Missing location information*
- *Missing statistics on people affected*
- *Notably, one instance of a missing epidemic 'name' (epidemic listed without a given disease) – this is shown in summary figure as an 'undefined' disease.*

"To compare the reporting consistencies across multiple open-access data sources, we extracted data for the same period from three additional open-access disaster databases / resources that report on natural disasters and epidemics or outbreaks, ReliefWeb, the WHO-DON and FloodList. "
and

"As a singular disaster report in one database could be considered the same as multiple data entries within another database, we calculated the proportion of disasters for each 'reference database' that could be considered the same event across all five database comparisons carried out. "
Can a sensitivity analysis on this approach, excluding possible duplicate entry across databases, please be considered?

Response: For this we have presented a concordance table highlighting the comparison between databases reporting the same hazards and disasters. For each database, we only used the first report of the hazard to avoid the duplication.

"To examine the relationship between natural disasters and subsequent epidemics, we examined the EM-DAT data to identify natural disasters that had occurred within 3 months prior to an epidemic in the same reported location (district)."
Can a sensitivity analysis on the choice of a 3 month window to assess this relationship please be considered?

Response: We have now included a form of sensitivity analysis based on the optimal transmission period of environmentally sensitive diseases such as cholera and dengue i.e., 0-3 months, and compared this the 4-6 month and 7–12-month periods. We examined the frequency of hazards as well as the number of people affected and included a new figure with two graphs to show these trends.

Can the authors please add a Statistical Analysis section to describe the methods they used to analyse the data?

"Figure 1. Summaries of EM-DAT January 2010 – September 2024 on the distribution of A) natural disasters subtype and B) epidemic subtype per country. "
and
"Figure 2. Summary of EM-DAT data January 2010 – September 2024. Graphs showing (A) the total number of people affected by each natural disaster and (B) the total number of people affected by each epidemic. "
Can distributions please also be shown and analysed over time?

Response: We have added in the related sections more information on the analyses that was conducted. Including the following

- *Summary statistics and Chi-square goodness of fit tests for the overall distribution of natural hazards by country, annually, monthly. This includes analysing the distributions of non-biological hazards and epidemics separately by hazard or disease type respectively.*

- *Chi-square goodness of fit tests to analyse the distribution of total people affected by country, non-biological natural hazards and epidemics separately.*

- *Summarised the entry numbers across all databases and summarised their cross-comparison statistics (what percentage of the compared databases matched).*

- *Identified the epidemics that occurred following natural hazards (based on 3 month rule) and summarised these (location, epidemic type, date, prior natural hazard) and compared to those with a great period of time between events. A one-way ANOVA test was performed to analyse differences in the total number of people affected by epidemics that occurred (1) within 3 months of a natural hazard (2) between 4 – 6 months after a natural hazard (3) between 7 – 12 months after a natural hazard.*

Can the Tables please be reformatted for improved readability (currently they overlap pages and are difficult to interpret)?

Response: This may be related to the submission process and their positions changing. We have now put the tables on individual pages to try to avoid such issues. We hope it is better this time.

"Open-access disaster database sources vary in the type of information they collect and have several data gaps, including limited georeferenced information, which restricts comparability and opportunities for detailed mapping and analysis. " [Strengths and Limitations of this data]
and
"We acknowledge that the use of open access data sources may have limitations if they are 221 incomplete as these sources most likely feature reporting biases and have not recorded all events. 222 We also acknowledge that our focus on a few selected data sources may cause unforeseen biases." [Data Limitations]
The authors suitably acknowledge the potential biases in the data.

Response: Done

Can the study limitations please be further addressed in the Discussion section of the article?

Response: We have added to aspects of the discussion to further highlight the limitations.

VERSION 2 - REVIEW

Reviewer 1

Name Egawa, Shinichi

Affiliation Tohoku University, International Cooperation for Disaster Medicine Lab.,International Research Institute of Disaster Science (IRIDeS)

Date 29-Sep-2025

COI

General comment

Thank you for the revision, which improved the readability.

The title can be more attractive to BMJ Open readers if you indicate a spatio-temporal analysis of “disasters and epidemics” rather than including everything under “natural hazards.” That may be the reason why the WHO named the Health Emergency and Disaster Risk Management (H-EDRM) Framework. Disaster includes “health emergency,” such as a pandemic. The strength of this paper lies in the fact that it found no correlation between the length of preceding ENSO phases. At the same time, it identified a possible causal relationship in 16 disasters that cascaded into epidemics within a particular interval. Discussing the factors that caused such cascades is a future research target.

The abstract should be much more attractive. You don’t have to exclude the climate from the title and abstract because it was discussed and serves as the starting point of this research. As you described in the Discussion section, climate change exacerbates food security issues and leads to internal displacement, ultimately contributing to social instability that may lead to armed conflicts. This is a vicious cycle. Preventing the epidemics requires good WASH and nutrition, as well as education. This study provides the statistical basis for a whole-of-society approach, as described in the H-EDRM Framework and the SDGs.

Then the statistics need to be reliable and relevant. Please see the point-by-point comments below.

I agree with the high variability of open-access databases because they serve individual purposes. Having EM-DAT as a reference is no problem because other databases are based on corrections of media content and updates from OCHA and other relief agencies. WHO-DON and FloodLists also correct various items. GLIDE serves as the reviewed provider of disaster identifiers, using a unique style agreed upon. Thus, both EM-DAT and ReliefWeb use GLIDE as an identifier, in addition to their serial numbers. You can find GLIDE numbers as External IDs when you download the Excel file from EM-DAT. I would appreciate it if you could label the disasters with the GLIDE numbers, especially in Table 5.

Point-by-point comments:

Line 44: The background of why and how you had the research question in the Greater Horn of Africa should be mentioned to make this abstract more attractive.

Line 44: "Africa" instead of "African."

Line 59: I recommend using "environment-sensitive diseases" or "climate-sensitive diseases" according to your research question and elaborated conclusion.

Lines 68: These are the strengths and limitations of "open-access databases." I recommend focusing on the strength of your study, which is the first cross-sectional analysis of natural hazard events and infectious disease relationships, while clarifying the lack of a standard database, warranting the improvement of databases. If you focus on climate change, describe its strengths, such as identifying no statistically significant correlation between the preceding length of ENSO phases and identifying 16 disasters that possibly cascaded into epidemics.

Line 72, Weakness: Where is "climate change?" Starting your Introduction section with climate change, the gaps between your research question, hypothesis, and your results should be mentioned as a weakness.

Line 257: "(NOAA)", Line 238: "NOAA."

Line 339: The number of affected people can be translated to per 100,000 background population of each country. A statistical comparison between countries is more meaningful when comparing the numbers per population.

Line 342: The uniform distribution hypothesis is valid only when the total population of the country is the same. I don't think this statistical comparison is meaningful, even if you divide the number of affected people by the total population, because the disaster risk to the community is not defined solely by the ratio of affected people.

Line 352: Express the number of affected people per population when you compare the countries.

Line 357: Use "natural hazard" instead of "natural disaster." Express the graph in n per population.

Line 384: Correct the n or percentage, if 130 were the same, 100%. if 52.0% is correct, n should be 67.6. or GLIDE n should be 250.

Line 389: If you want to emphasize the difference in reporting, summarize the difference (ex., number of affected people as of the reported date). By which, you have to convince the readers that EM-DAT deserves as a reference database. In case 1, EM-DAT indicates that 1.3 million people are affected, while ReliefWeb (GLIDE) reports 600 or more people, based on the May 17 IFRC report. Please confirm whether the number of affected people in the EM-DAT refers to infected patients or simply the total affected community population, as EM-DAT indicates that vaccination was conducted.

Line 396: In case 2, ReliefWeb reported 20 deaths (13 adults, seven children), 269 people injured, and 1,170 people in total who were affected, which is double that of EM-DAT. Clarify what you want to say by using these examples.

Lines 394 and following examples: The URLs do not link to the correct disasters. As you can see in the URL of ReliefWeb, their list is using the GLIDE numbers (EP-2021-000047-KEN) to identify the disaster. The line for GLIDE is unnecessary.

Line 407: The number of the affected people in EM-DAT and ReliefWeb is close to 900,000 on Oct. 13. The GLIDE and FloodList report 90,000 on Aug. 8. This discrepancy suggests the difficulty of correctly reporting and evaluating the disaster impact.

Line 415: If you search "TC-2018-000059-SOM" on the GLIDE system, you can get one result:
https://glidenumber.net/glide/public/search/details.jsp?glide=21068&record=1&last=1. The number of affected people is not well described in other databases.

Line 417: By this drought, ReliefWeb reports the estimation of FAO as 12.9M people (FAO WFP 23 Mar 2021) as the most significant number within the lengthy report. The ReliefWeb classifies food crisis levels for prioritizing humanitarian relief. As you see, it is not easy to define the number of affected people. Hence, I would appreciate it if you could elaborate on the risks associated with relying on a single database.

Line 432: Both EM-DAT and ReliefWeb are using the GLIDE number to identify the disaster, although several disasters are not registered on GLIDE.

Lines 473 and 476: Use "natural hazard"

Line 488: Clarify that these average lengths are preceding the disasters.

Table 5, title: Clarify that the average length is "preceding the disasters." Add the raw total to show the overall average length of three phases is 8 months, respectively.

Line 509: Delete "especially natural disasters" because you did not include any human-induced hazards.

Line 510: It is unclear why "millions of people" are vulnerable. "Millions of people" is fine.

Line 549: Use "natural hazards" or "disasters."

Line 559: As mentioned above, the 1.3M people affected by Dengue in 2022 in Kenya may include the people vaccinated. Add explanations and discussions.

Line 578: Correct "and for."

Supplementary file 8 (5?): Use "natural hazard onset month" or "disaster onset month."

**Reviewer**	**2**
**Name**	**Kelson, Zoe**

**Affiliation**	**University of Exeter, Mathematics**
**Date**	**30-Sep-2025**
**COI**	

Thanks to the authors for their responses and revisions.

The following reviewer requests are raised:

Author response:

"Can confirm that data was missing in our primary database EM-DAT. These are also evident in the database provided – supplemental file 2. Examples of missing data that restricted some of the analysis included: • Incomplete start/end dates (missing month – 10 disasters had no start month listed) • Missing location information • Missing statistics on people affected • Notably, one instance of a missing epidemic 'name' (epidemic listed without a given disease) – this is shown in summary figure as an 'undefined' disease ."

Reviewer request:

Whilst missing data is acknowledge in the Discussion, can the authors please further incorporare the above response to reviewer into the article?

Author response:

"A one-way ANOVA test was performed to analyse differences in the total number of people affected by epidemics that occurred (1) within 3 months of a natural hazard (2) between 4 – 6 months after a natural hazard (3) between 7 – 12 months after a natural hazard."

Reviewer request:

Can the authors please confirm if and how normality was verified for the application of this parametric methodology?

VERSION 2 - AUTHOR RESPONSE

Reviewer: 1
Dr. Shinichi Egawa, Tohoku University
Comments to the Author:
General comment
Thank you for the revision, which improved the readability.
The title can be more attractive to BMJ Open readers if you indicate a spatio-temporal analysis of "disasters and epidemics" rather than including everything under "natural hazards." That may be the reason why the WHO named the Health Emergency and Disaster Risk Management (H-EDRM) Framework. Disaster includes "health emergency," such as a pandemic. The strength of this paper lies in the fact that it found no correlation between the length of preceding ENSO phases. At the same time, it

identified a possible causal relationship in 16 disasters that cascaded into epidemics within a particular interval. Discussing the factors that caused such cascades is a future research target.

Response. We have modified the title in line with our recent revisions and your suggestion.

The abstract should be much more attractive. You don't have to exclude the climate from the title and abstract because it was discussed and serves as the starting point of this research. As you described in the Discussion section, climate change exacerbates food security issues and leads to internal displacement, ultimately contributing to social instability that may lead to armed conflicts. This is a vicious cycle. Preventing the epidemics requires good WASH and nutrition, as well as education. This study provides the statistical basis for a whole-of-society approach, as described in the H-EDRM Framework and the SDGs.
Then the statistics need to be reliable and relevant. Please see the point-by-point comments below.

Response: We have adhered to the journal format and reported as much as we can within the word limit and added more on the relationship between hazards and epidemics to make it more attractive.

I agree with the high variability of open-access databases because they serve individual purposes. Having EM-DAT as a reference is no problem because other databases are based on corrections of media content and updates from OCHA and other relief agencies. WHO-DON and FloodLists also correct various items. GLIDE serves as the reviewed provider of disaster identifiers, using a unique style agreed upon. Thus, both EM-DAT and ReliefWeb use GLIDE as an identifier, in addition to their serial numbers. You can find GLIDE numbers as External IDs when you download the Excel file from EM-DAT. I would appreciate it if you could label the disasters with the GLIDE numbers, especially in Table 5.

Response: We have added GLIDE numbers to the revised Table 4. All other related GLIDE numbers are listed in supplementary tables. We have indicated in Table 4 where GLIDE IDs could not be linked directly from the GLIDE database download (GLIDE not available). We acknowledge that current EM-DAT downloads provide GLIDE numbers in the "External IDs" column, however downloading this data now for the countries in our study shows that EM-DAT features many missing GLIDE IDs,

corroborating our findings that many EM-DAT disasters cannot be linked to disasters in the GLIDE database.

Point-by-point comments:
Line 44: The background of why and how you had the research question in the Greater Horn of Africa should be mentioned to make this abstract more attractive.

Response: We have limited space to add this in but the rational in in the introduction and methods.

Line 44: "Africa" instead of "African."
Response: Amended.

Line 59: I recommend using "environment-sensitive diseases" or "climate-sensitive diseases" according to your research question and elaborated conclusion.

Response: We have modified to include climate-and environmentally sensitive diseases, in addition to the abovementioned comments.

Lines 68: These are the strengths and limitations of "open-access databases." I recommend focusing on the strength of your study, which is the first cross-sectional analysis of natural hazard events and infectious disease relationships, while clarifying the lack of a standard database, warranting the improvement of databases. If you focus on climate change, describe its strengths, such as identifying no statistically significant correlation between the preceding length of ENSO phases and identifying 16 disasters that possibly cascaded into epidemics.

Response: We have included an additional strength related to the analysis of the various relationships.

Line 72, Weakness: Where is "climate change?" Starting your Introduction section with climate change, the gaps between your research question, hypothesis, and your results should be mentioned as a weakness.
Response: The reference to climate change in the introduction was to provide context. We do not see this a as limitation of the study as our aim was not examine climate change in relation to the data. To add in in here would be confusing as it is not the role of the databases.

Line 257: "(NOAA)", Line 238: "NOAA."

Response: Amended.

Line 339: The number of affected people can be translated to per 100,000 background population of each country. A statistical comparison between countries is more meaningful when comparing the numbers per population.

Response: The geographical location and population denominators of each hazard and disaster are not known or very difficult to define and quite variable between reported, therefore the rates per population may be misleading and we advocate to only state the numbers of affected people as reported by EM-DAT.

Line 342: The uniform distribution hypothesis is valid only when the total population of the country is the same. I don't think this statistical comparison is meaningful, even if you divide the number of affected people by the total population, because the disaster risk to the community is not defined solely by the ratio of affected people.

Response: We have excluded the comparison between countries.

Line 352: Express the number of affected people per population when you compare the countries.
Response: As noted above re rates.

Line 357: Use "natural hazard" instead of "natural disaster." Express the graph in n per population.
Response: Amended.

Line 384: Correct the n or percentage, if 130 were the same, 100%. if 52.0% is correct, n should be 67.6. or GLIDE n should be 250.

Response: Amended.

Line 389: If you want to emphasize the difference in reporting, summarize the difference (ex., number of affected people as of the reported date). By which, you have to convince the readers that EM-DAT deserves as a reference database. In case 1, EM-DAT indicates that 1.3 million people are affected, while ReliefWeb (GLIDE) reports 600 or more people, based on the May 17 IFRC report. Please confirm whether the number of affected people in the EM-DAT refers to infected patients or simply the total affected community population, as EM-DAT indicates that vaccination was conducted.

Response: The purpose of this section is to highlight the variability between the various databases. The first section of each example clearly highlights the range of data reported by EM-DATA including the number of people affected.

Line 396: In case 2, ReliefWeb reported 20 deaths (13 adults, seven children), 269 people injured, and 1,170 people in total who were affected, which is double that of EM-DAT. Clarify what you want to say by using these examples.

Response: As noted above, the purpose of this section is to highlight the variability between the various databases. It is beyond the scope to report every detail that each database reports.

Lines 394 and following examples: The URLs do not link to the correct disasters. As you can see in the URL of ReliefWeb, their list is using the GLIDE numbers (EP-2021-000047-KEN) to identify the disaster. The line for GLIDE is unnecessary.
Line 407: The number of the affected people in EM-DAT and ReliefWeb is close to 900,000 on Oct. 13. The GLIDE and FloodList report 90,000 on Aug. 8. This discrepancy suggests the difficulty of correctly reporting and evaluating the disaster impact.

Response: We have found that quite a few errors arose when we tried to link the GLIDE numbers. We have re-added in the GLIDE links.

Line 415: If you search "TC-2018-000059-SOM" on the GLIDE system, you can get one result: https://glidenumber.net/glide/public/search/details.jsp?glide=21068&record=1&last=1. The number of affected people is not well described in other databases.

Response: Link added.

Line 417: By this drought, ReliefWeb reports the estimation of FAO as 12.9M people (FAO WFP 23 Mar 2021) as the most significant number within the lengthy report. The ReliefWeb classifies food crisis levels for prioritizing humanitarian relief. As you see, it is not easy to define the number of affected people. Hence, I would appreciate it if you could elaborate on the risks associated with relying on a single database.
Response: We have addressed this is the discussion.

Line 432: Both EM-DAT and ReliefWeb are using the GLIDE number to identify the disaster, although several disasters are not registered on GLIDE.

Response: The table highlights the range of discrepancies between the different databases.

Lines 473 and 476: Use "natural hazard"

Response: Amended.

Line 488: Clarify that these average lengths are preceding the disasters.

Response: Added.

Table 5, title: Clarify that the average length is "preceding the disasters." Add the raw total to show the overall average length of three phases is 8 months, respectively.

Response: Added

Line 509: Delete "especially natural disasters" because you did not include any human-induced hazards.

Response: Amended

Line 510: It is unclear why "millions of people" are vulnerable. "Millions of people" is fine.

Response: Amended.

Line 549: Use "natural hazards" or "disasters."

Response: Amended.

Line 559: As mentioned above, the 1.3M people affected by Dengue in 2022 in Kenya may include the people vaccinated. Add explanations and discussions.

Response: More information has been added to consider previous exposure, levels of immunity and vaccination rates where applicable.

Line 578: Correct "and for."

Response: Amended.

Supplementary file 8 (5?): Use "natural hazard onset month" or "disaster onset month."

Response: This is the correct number of the supplementary file.

Reviewer: 2
Prof. Zoe Kelson, University of Exeter
Comments to the Author:
Thanks to the authors for their responses and revisions.
The following reviewer requests are raised:

Author response:
"Can confirm that data was missing in our primary database EM-DAT. These are also evident in the database provided – supplemental file 2. Examples of missing data that restricted some of the analysis included: • Incomplete start/end dates (missing month – 10 disasters had no start month listed) • Missing location information • Missing statistics on people affected • Notably, one instance of a missing epidemic 'name' (epidemic listed without a given disease) – this is shown in summary figure as an 'undefined' disease ."
Reviewer request:
Whilst missing data is acknowledge in the Discussion, can the authors please further incorporate the above response to reviewer into the article?

Response: We have added to what we have already included in the discussion to make it more complete.

Author response:
"A one-way ANOVA test was performed to analyse differences in the total number of people affected by epidemics that occurred (1) within 3 months of a natural hazard (2) between 4 – 6 months after a natural hazard (3) between 7 – 12 months after a natural hazard."
Reviewer request:
Can the authors please confirm if and how normality was verified for the application of this parametric methodology?

Response: We have amended this ANOVA test to a Mann-Whitney U test following confirmation that data for the total people affected was not normally distributed. We used the Mann-Whitney U test to compare the distribution of the total number

of people affected between only two transmission periods: (1) 0-3 months and (2) the combined 4-12 months.
